# The Implications of Artificial Intelligence in Pedodontics: A Scoping Review of Evidence-Based Literature

**DOI:** 10.3390/healthcare12131311

**Published:** 2024-06-30

**Authors:** Salvatore La Rosa, Vincenzo Quinzi, Giuseppe Palazzo, Vincenzo Ronsivalle, Antonino Lo Giudice

**Affiliations:** 1 Section of Orthodontics, Department of Medical-Surgical Specialties, School of Dentistry, University of Catania, Via Santa Sofia 78, 95123 Catania, Italy; gpalazzo@unict.it (G.P.); antonino.logiudice@unict.it (A.L.G.); 2 Department of Life, Health & Environmental Sciences, Postgraduate School of Orthodontics, University of L’Aquila, 67100 L’Aquila, Italy; 3 Section of Oral Surgery, Department of General Surgery and Medical-Surgical Specialties, School of Dentistry, Policlinico Universitario “Gaspare Rodolico—San Marco”, University of Catania, Via Santa Sofia 78, 95123 Catania, Italy; vincenzo.ronsivalle@hotmail.it

**Keywords:** artificial intelligence (AI), pediatric dentistry, pedodontics

## Abstract

Background: Artificial intelligence (AI) has emerged as a revolutionary technology with several applications across different dental fields, including pedodontics. This systematic review has the objective to catalog and explore the various uses of artificial intelligence in pediatric dentistry. Methods: A thorough exploration of scientific databases was carried out to identify studies addressing the usage of AI in pediatric dentistry until December 2023 in the Embase, Scopus, PubMed, and Web of Science databases by two researchers, S.L.R. and A.L.G. Results: From a pool of 1301 articles, only 64 met the predefined criteria and were considered for inclusion in this review. From the data retrieved, it was possible to provide a narrative discussion of the potential implications of AI in the specialized area of pediatric dentistry. The use of AI algorithms and machine learning techniques has shown promising results in several applications of daily dental pediatric practice, including the following: (1) assisting the diagnostic and recognizing processes of early signs of dental pathologies, (2) enhancing orthodontic diagnosis by automating cephalometric tracing and estimating growth and development, (3) assisting and educating children to develop appropriate behavior for dental hygiene. Conclusion: AI holds significant potential in transforming clinical practice, improving patient outcomes, and elevating the standards of care in pediatric patients. Future directions may involve developing cloud-based platforms for data integration and sharing, leveraging large datasets for improved predictive results, and expanding AI applications for the pediatric population.

## 1. Introduction

Although John McCarthy introduced the term “Artificial Intelligence” (AI) in a 1956 conference, the concept itself goes back to 1943, with a work by McCulloch and Pitts. The goal was to build automated devices, also called machines, capable of carrying out human-level tasks in the field of informatics and mathematics [1]. While it might be challenging to define, AI is generally understood to refer to a machine program that is able to think and perform cognitive activities [2,3]. AI is applied in many different fields these days, such as economics, video games, cellphones, healthcare, and the auto industry [4]. It is critical to get a solid grasp of fundamental AI terminology in order to completely appreciate the implications of AI on the subject of pedodontics, as reported in Table 1.

Artificial intelligence has quickly gained traction in science and technology. It mostly depends on imaging, which forms the foundation of dentistry. Furthermore, AI is very helpful in evaluating and tracking a patient’s health over time, comprehending the long-term effects of medication, and foreseeing potential health risks [10,13]. Artificial intelligence has the potential to replace the long hours worked by dental practitioners. Moreover, in pedodontics, its first usage was to identify cephalometric landmarks in an automatized way, without human help.

It is also conceivable to integrate healthcare for all, improve people’s health at a reduced cost, and deliver individualized, preventative, and predictive dentistry. AI may, above all, raise the bar for dental care by optimizing diagnosis efficacy and accuracy, enhancing treatment visualization, simulating results, and forecasting oral health and disorders [7,10,13].

Consideration has also been given to utilizing AI models as additional tools to improve the precision and accuracy of diagnostics. Artificial intelligence (AI) has found widespread application in the medical sciences and has demonstrated considerable success in various aspects of patient care. This encompasses evaluating a patient’s likelihood of falling ill and detecting various diseases [14,15,16].

This scoping review aims to comprehensively investigate the existing body of literature concerning the integration of AI in pediatric dentistry. The focus is on delving into recent advancements and the transformative possibilities that this technology holds, with a specific emphasis on its potential to enhance dental health outcomes for children.

## 2. Materials and Methods

The present scoping review will follow the guidelines of the Joanna Briggs Institute (JBI) for scoping reviews and the Preferred Reporting Items for Systematic Reviews and Meta-Analyses (PRISMA) extensions for scoping reviews (PRISMA-ScR) [17,18]. The protocol of this scoping review is available as Supplementary Material S1, and it is registered in the Open Science Framework database (https://osf.io/z6prb) from 18 June 2024.

### 2.1. Research Question

We developed a key research question to guide our search for strategies. The answer to this question could be used by pediatric dentists as a reference: “What are the current developments and challenges in the application of artificial intelligence in pediatric dentistry?”.

### 2.2. Eligibility Criteria

This review selectively incorporates original research articles that delve into the application of AI models in the realms of pediatric dentistry and pedodontics. To maintain focus and rigor, incomplete texts, scoping reviews, narrative reviews, case series, consensus conferences, and articles written in languages other than English have all been excluded from consideration, as well as studies including adults. Moreover, there was no restriction on the year of publication of the studies. This meticulous approach ensures a thorough examination of substantive and pertinent research contributions in the specified field.

### 2.3. Literature Sources and Search Parameters

To assess the corpus of the current literature on the subject, a few database searches were carried out from July 2023 through December 2023. The development of a search strategy that incorporated all discovered keywords and free-standing terms was aided by a health sciences librarian. The Web of Science, Embase, Scopus, and PubMed databases were utilized. To confirm the validity of every source of evidence listed in the reference list, additional research was looked into. The outcomes of modifying the search approach for every database are shown in Supplementary Table S1.

### 2.4. Data Cleaning

#### 2.4.1. Study Selection

Following the acquisition of search results from every electronic database, the citations were imported into EndNote X9, a reference manager program developed by Clarivate^TM^, London, UK. Reports that were duplicates were eliminated, and articles that provided updates or preliminary findings were only assessed once. Two writers, A.L.G. and S.L.R., checked all of the titles and abstracts they had gathered from the databases before reading the full texts of any relevant studies. The eligibility of the studies was evaluated objectively, and any disagreements were settled after discussion with another author, G.P. The agreement between the reviewers was highly reliable, with a kappa value of 0.963.

#### 2.4.2. Data Extraction

In order to gather the characteristics and outcomes (study design, sample size, and objectives) needed for the ensuing literature analysis, two authors (S.L.R. and A.L.G.) created a data extraction form. We discussed any discrepancies with G.P., another author reviewer. Cohen’s kappa statistics were employed to evaluate the degree of concurrence between the two authors, resulting in a value indicating high reliability, 0.963.

### 2.5. Information Synthesis

The methodology employed in reporting the findings of this study was derived from established frameworks delineated in prior research conducted by other scholars [14,19]. In order to enhance the relevance to clinical indications, the observations were systematically categorized and discussed within distinct domains. These domains were meticulously structured to encompass all the pertinent data extracted from the studies incorporated in the analysis. This approach ensures a systematic and comprehensive presentation of the research outcomes, building upon the foundations laid by previous scholarly investigations.

## 3. Results

### 3.1. Study Inclusion (Study Characteristics)

**Figure 1:**
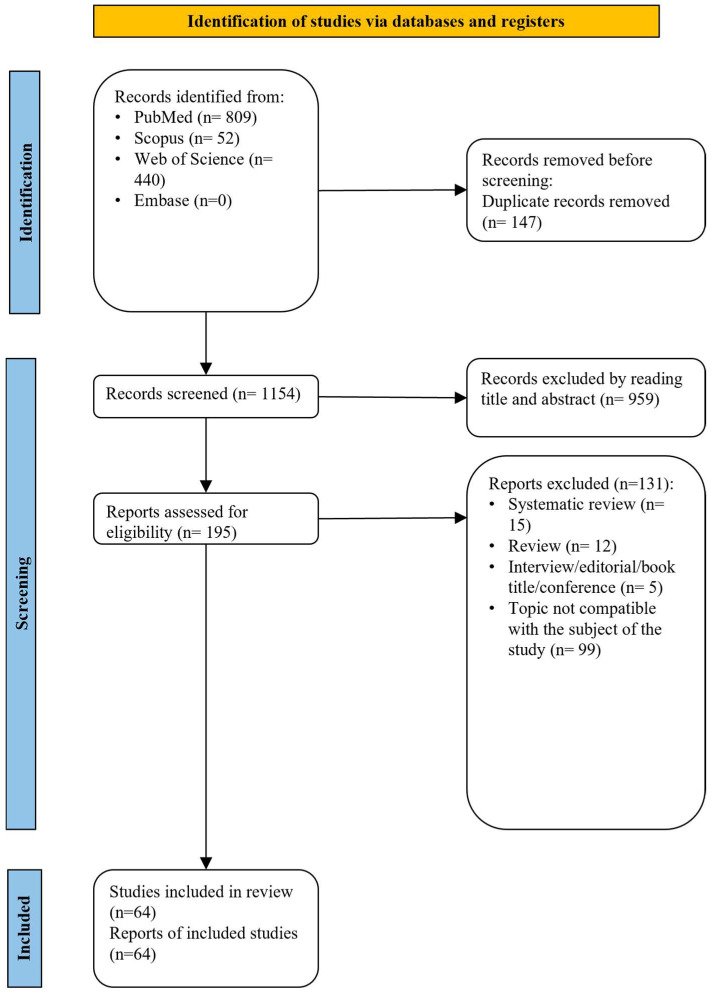
Flow-chart of the study selection.

The reviewers examined 1154 records out of the 1301 citations that the strategy searches had turned up after removing duplicate files. Following the first screening of abstracts and titles, 959 articles were deemed unacceptable. The full texts of the remaining 195 articles were then obtained for additional review. After a comprehensive examination of the entire texts of those papers, 64 studies were judged to be appropriate for the review. The publications that were eliminated at this time are included in Supplementary Table S2, along with the reasons given. Figure 1 provides a summary of all study selection information. Table 2 contains details of all the included articles.

### 3.2. Study Characteristics

The primary areas of focus for AI models created for use in pediatric dentistry have been the following: orthodontic diagnosis (n = 3) [57,64,72], automated cephalometric tracing (n = 9) [35,45,48,49,53,56,58,60,68], segmentation and landmark identification (n = 7) [22,23,27,39,52,71,80], AI-driven remote monitoring (n = 2) [33,34], estimation of growth and development (n = 11) [21,36,41,43,47,48,55,65,66,67,79], dental plaque and cavities (n = 4) [30,62,74,75], evaluating pediatric oral health through toolkits developed by machine learning (n = 3) [8,31,73], supernumerary tooth identification (n = 8) [2,11,20,29,32,42,46,54], early childhood caries (ECC) (n = 7) [37,44,59,61,63,69,78], chronological age assessment (n = 6) [25,28,51,70,76,77], identification of deciduous and young permanent teeth (n = 6) [24,26,28,38,40,50].

## 4. Discussion

**Figure 2:**
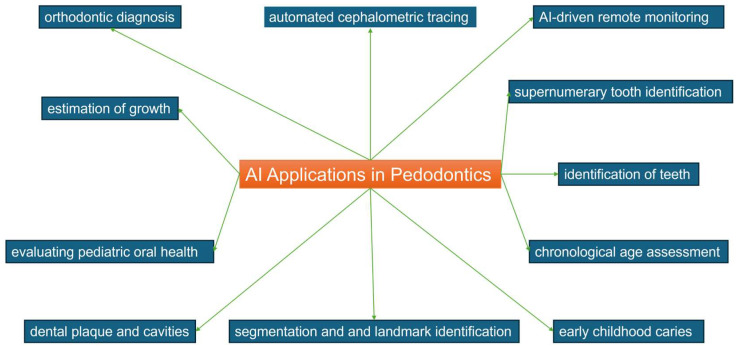
Field of application of AI in pedodontics.

The integration of AI technology into dental practices holds the potential to elevate the standard of dental treatment significantly. By providing support to dentists, AI facilitates optimal dental care, leading to enhanced accuracy in diagnostics, treatment planning, and outcome predictions. Notably, deep learning plays a pivotal role in diagnosis, promising increased productivity, and improved precision across dental procedures. The advent of readily available data has allowed AI to demonstrate its efficacy in various pediatric dentistry applications, with Convolutional Neural Network (CNN) models proving particularly effective in expediting and refining patient diagnoses. This collaborative approach encourages active patient engagement, thereby contributing to the overall success rate of dental care [19,81,82,83]. Figure 2 explores the field of the application of AI in pedodontics.

### 4.1. Orthodontic Diagnosis

Accurate and precise orthodontic diagnosis relies on patient data, which is meticulously collected from a comprehensive database containing a detailed inventory of the patient’s concerns. This orthodontic diagnostic database is compiled through various means, such as written or verbal interviews, clinical examinations, and a thorough examination of patient records, including dental impressions, radiographs, and diagnostic photographs [84]. The patient assessment process in clinical settings faces challenges related to both accuracy and time constraints. Recognizing the need for enhanced efficiency, particularly in imaging and diagnosis, automation has become imperative given the substantial time investment required for comprehensive patient evaluations and record compilation [85]. Orthodontic diagnosis poses unique challenges, demanding a thorough evaluation of various facial structures. The transition to digital platforms for patient data collection, coupled with the establishment of digital databases for diagnostic and treatment purposes, has been facilitated by the integration of digital dentistry tools. While digital data acquisition has expedited the diagnosis and treatment phases, the analysis and decision-making stages still rely on the expertise of a clinician. This combination of automation and clinician experience aims to strike a balance, ensuring both speed and accuracy in the orthodontic assessment process [86,87]. Evaluation workloads have been greatly decreased and diagnostic variation has been minimized thanks to automation systems that use AI and ML technologies [85]. Orthodontic research has looked at a number of AI algorithms, all of which relied on voluminous patient examination records as input data. Some studies revealed that the integration of artificial intelligence into the diagnostic process resulted in a reduced dependency on specialized medical professionals and a decreased likelihood of incorrect diagnoses. The promising outcomes led researchers to conclude that the application of AI holds substantial potential within the field of orthodontics. The findings suggest that leveraging AI technology can not only streamline the diagnostic process but also contribute to increased accuracy, marking a significant advancement in orthodontic practices [57,72,85,86,88,89].

A separate study has put forth findings suggesting that machine learning exhibits the potential to elevate orthodontic diagnosis and treatment planning. The study concludes that by anticipating linear dental arch measurements and proactively addressing anterior segment malocclusion, machine learning can significantly contribute to improved accuracy in orthodontic assessments and treatment planning. The high accuracy demonstrated in the study highlights the promising prospects of integrating machine learning into orthodontic practices for more effective and precise outcomes [64].

### 4.2. Automated Cephalometric Tracing

In the realm of pediatric orthodontics, the diagnosis and treatment planning processes heavily rely on cephalometry—a technique that measures soft tissue profiles, facial features, and skull bones. Cephalometric tracing, a pivotal aspect of this methodology, can be executed using either computer software or manual labor [53,68,90]. The process of manual cephalometric tracing is laborious and subject to human error in identifying landmarks and measuring cephalometric parameters. The primary mistakes in manual tracing are frequently related to how radiographs can be unpredictable when it comes to measuring and identifying landmarks [48,56]. Efficiencies in time and accuracy are realized through the adoption of computer-assisted cephalometric tracing, minimizing the potential for human error and enhancing the diagnostic precision of cephalometric analysis [45]. Earlier research articles indicate that the integration of AI-driven automated cephalometric tracing yields positive outcomes, having achieved a notably high success rate surpassing 90%, particularly in the differentiation of cephalometric landmarks using computerized software and web-based applications [35,49,53,58]. This innovative technique harnesses artificial intelligence and employs cutting-edge deep learning methods to identify cephalometric landmarks, aiming to reduce human error and enhance overall time efficiency in orthodontic procedures [60].

### 4.3. Segmentation and Landmark Identification

In medical image analysis, image segmentation is an essential procedure that involves separating pixels in X-rays, CT scans, or MRI images that correspond to specific organs or lesions [91]. Volumetric medical image analysis and computer-aided diagnosis systems heavily rely on precise segmentation, making the identification of landmarks in lateral cephalometric X-rays crucial for diagnosis and treatment planning in orthodontics [92]. Several investigations have targeted the automation of landmark identification in lateral cephalometric X-rays [22,35,45,58,60,93]. The introduction of Convolutional Neural Networks (CNNs) has been pivotal in this endeavor. Arik [22] was among the pioneers to employ CNNs for automated landmark identification, while Park and Hwang [35,60] utilized You-Only-Look-Once version 3 (YOLOv3) as their deep learning method of choice. Kunz [45] successfully automated the identification of 12 commonly used orthodontic parameters using an open-source CNN. Nishimoto [58] utilized CNNs to automatically identify landmarks, utilizing lateral cephalometric radiographs from diverse sources such as the internet and personal computers. These advancements underscore the transformative potential of deep learning in enhancing the accuracy and efficiency of orthodontic image analysis and diagnosis.

According to Bag’s study [23], automatic segmentation of anatomical landmarks with YOLOv5 in orthopantomographs, such as the orbit, maxillary sinus, mandibular canal, mental foramen, foramen mandible, incisura mandible, articular eminence, and condylar and coronoid processes, may accelerate clinical diagnosis for doctors and raise their awareness of pathologies related to these structures. The orbit, maxillary sinus, mandibular canal, incisura mandible, and condylar process yielded the best sensitivity levels. The articular eminence (0.92) and mental foramen (0.92) had the lowest sensitivity ratings.

Kaya et al. [39] conducted a study utilizing YOLOv4, an object detection model based on Convolutional Neural Networks (CNNs), to analyze the orthopantomographs of children aged 5 to 13. The study demonstrated excellent results in the detection of immature teeth, permanent tooth germs, and brackets. However, certain dental procedures and structures, such as fillings, root canal therapy, and extra teeth, were not recognized effectively. Despite these limitations, employing a deep learning-based method for the identification of specific dental structures and past dental treatments in pediatric panoramic X-rays holds great potential. This approach may assist dentists in the early diagnosis of dental defects and enable more precise treatment options, ultimately saving time and effort in the pediatric dental care landscape.

Wang et al. created an automated cone beam computed tomography (CBCT) method for maxilla and mandibular segmentation [71]. They segmented both structures at the same time using a learning-based framework based on random forest [94,95]. The dice ratio, which calculates the overlap between the segmented and ground truth sets, is frequently used to assess volumetric segmentation in medical images.

In order to examine changes in maxillary structure in response to unilateral canine impaction, Chen et al. [27] improved Wang’s method [71]. The segmentation process was much more efficient thanks to the automatic algorithm.

Using a CNN deep learning algorithm, Lo Giudice et al. [52,96] and Leonardi et al. [97] have separated the jaw and airways using artificial intelligence, respectively.

Another study conducted by Gillot et al. [80] proposed an automated landmark identification method in CBCT images through the usage of the ALICBCT algorithm, an AI source. In this method, there is a first phase of machine training, followed by the usage of AI to identify landmark positions, which is faster than the clinician’s identification, with high accuracy.

### 4.4. AI-Driven Remote Monitoring

Harnessing the power of smartphones, patients or their parents can now employ artificial intelligence-driven remote monitoring (AIRM) to independently scan and record their dentition. This innovative approach empowers clinicians with the capability to remotely monitor patients’ oral health. Deep learning (DL) is employed by AIRM to improve tooth-movement tracking and identify key features from patient images. Research has delved into the accuracy of this technology and its consequential impact on treatment, showcasing the transformative potential of AI-driven remote monitoring in the realm of dental care.

According to a pilot study, patients felt generally good about their experience with Invisalign, and AIRM may help patients need fewer treatments [98,99,100]. According to a recent study [33], the use of AI-driven remote monitoring (AIRM) reduced the number of patient appointments by about 3.5 visits, or 33.1%. Nevertheless, no clinically significant differences in the course of treatment or the advancement of dental alignment were found in the study.

The main benefit of AIRM is its capacity to reduce in-office visits, providing patients and their parents with more flexibility and convenience during their course of treatment [34]. Additionally, it creates a forum for improved patient–doctor communication. However, factors including the pedodontist’s practice, patient demographics, and geographic location may affect how cost-effective AIRM is overall [34].

Remote monitoring has emerged as a valuable technique in orthodontic therapy, enabling practitioners to conduct more frequent patient assessments while minimizing the necessity for extended in-person visits [101]. The incorporation of this technology has led to notable improvements in patient hygiene, precise diagnosis, and treatment planning. This modernization of the therapeutic process has not only streamlined patient care but has also significantly enhanced overall treatment outcomes in orthodontics.

### 4.5. Estimation of Growth and Development

In orthodontic treatment, precise timing is crucial, and anthropometric indices such as chronological age, menarche, vocal changes, height increase, and skeletal maturation (skeletal age) serve as valuable parameters for assessing growth and development [102,103]. Recognizing the substantial variability in growth dynamics during adolescence among individuals, relying solely on chronological age proves insufficient for estimating the remaining growth potential [41,104]. Skeletal age assessment becomes more appropriate for evaluating individual growth [105,106,107,108], often determined through radiographs that reveal signs of skeletal maturity [109].

The cervical vertebral maturation (CVM) approach, frequently utilized for radiological skeletal maturation measurement, involves identifying growth-related changes in wrist bones or vertebral bodies [110,111]. The CVM approach demonstrates a strong correlation with the assessment of skeletal maturity through wrist X-rays [112,113,114]. Given the inherent role of lateral cephalometric radiographs in orthodontic diagnostics, they offer a radiation-free alternative for evaluating vertebral body maturation in teenage patients [79,115,116,117]. The CVM technique focuses on morphological changes in vertebral bodies C2–C4, categorized into six stages corresponding to skeletal maturity [118]. However, accurate assessment can be challenging for inexperienced practitioners and prone to inaccuracy due to individual variations [41,65,119,120,121].

Recent advancements leverage artificial intelligence (AI) to automate the skeletal age determination process, providing an objective and standardized approach to overcome challenges associated with manual assessment [21,36,43,47,48,55,66,67,79]. This integration of AI holds promise in enhancing the accuracy and efficiency of skeletal age evaluation in orthodontics.

### 4.6. Dental Plaque and Cavities

Dental plaque is characterized as a bacterial community primarily adhering to the gingival margins and interproximal areas, subsequently extending to the teeth [74]. Its identification can pose challenges, particularly in minimal quantities, where distinguishing between the tooth and the plaque becomes intricate. Historically, practitioners utilized explorers or revealing solutions to mark affected areas, although these methods proved to be time-consuming and discomforting. Other side effects include an unpleasant taste and prolonged discoloration on the lips and oral tissue, which are primarily cosmetic in nature. While alternative approaches employ digital image analysis and autofluorescence spectroscopy, they are constrained by technological and cost-related limitations [122,123,124]. With the development of digital cameras and image analysis software, the first practical imaging technique that assessed the full plaque-affected region, if any was present, was presented [125,126].

However, using DL techniques, some research has been conducted to identify primary or permanent teeth that are impacted by plaque [74,75]. One study developed CNN models that can recognize plaque accumulation based on 886 dental pictures. Performance levels that were clinically acceptable were demonstrated by comparing the model to the analysis of a pediatric dentist with training. There are some disadvantages as well, since the final image quality greatly influenced the results, and the AI’s rationales for employing different methods to recognize plaque were not entirely obvious [74]. When these limitations are removed, both doctors and parents may employ AI technology to regularly check on their children’s dental health. An intraoral camera was used to take pictures, and 440 images were used to train the DL system, providing permanent-tooth plaque detection [75]. The technique demonstrated its ability to identify dental plaque placed on permanent teeth; nevertheless, the accuracy of the plaque identification decreased with increasing complexity of the dental plaque marginal line and dental plaque percentage.

The use of dental images to identify caries and molar–incisor hypomineralization (MIH) was assessed in a different research study [30]. The model achieved excellent accuracy for the pixel-by-pixel detection and localization of MIH and caries. However, not all dental conditions or treatments that impact teeth because of genetics or development have been included in the model so far. Furthermore, only really good photos were considered. In order to assess the suitability of VGG19, a deep learning method, as an additional tool and to determine the accuracy with which a convolutional neural network (CNN) can identify healthy teeth and early carious lesions on occlusal surfaces, the use of a digital camera for caries detection was proposed [62]. Both the primary and secondary identification of early carious lesions yielded satisfactory results using this strategy. This means that it can improve the accuracy of disease detection and serve as a valuable tool for the fast detection of caries lesions, enabling the formation of more prompt and reliable clinical judgments.

### 4.7. Evaluating Pediatric Oral Health through Toolkits Developed by Machine Learning

In contrast to other facets of general well-being, dental health frequently suffers, particularly in emerging and disadvantaged nations. A questionnaire on oral health was created by the World Health Organization (WHO) [73] for both adults and children in recognition of this gap. By creating machine learning-based oral health assessment toolkits that can precisely predict the Children’s Oral Health Status Index (COHSI) and Referral for Treatment Needs (RFTN), a research team hopes to solve these issues [73,127].

In the second study [127], the team utilized the PROMIS framework to create a conceptual model, known as the oral health item bank system. This model, designed by a multidisciplinary team of specialists, including PROMIS experts, general dentists, pediatric dentists, and social scientists, was anchored on three pillars: social, mental, and physical health. The resulting oral health item bank system demonstrated versatility, expanding its applicability beyond the realm of oral health assessments. It now encompasses the creation of tailored short forms designed for program evaluation and the formulation of oral health policies.

A toolkit has been devised, featuring a concise assessment form (SF) that incorporates dimensions related to physical, mental, and social health. This resource is designed to assist parents in evaluating their children’s oral health and identifying potential treatment needs [73]. The accuracy of this toolkit was influenced by factors such as the posed questions, the level of understanding by parents and children, the timing of survey completion, and, notably, the creation of an ML algorithm. Although the toolkit was created to support dentists in their examinations, it was not meant to be a substitute for physical examinations. The toolkit assigned rankings to participants according to percentiles, evaluated their overall oral health status, and determined the need for any necessary therapeutic interventions.

In a different study project [8], the relationship between teenage quality of life and dental health was investigated using statistical techniques and artificial intelligence algorithms.

In a separate research endeavor, statistical methods and artificial intelligence algorithms were employed to explore the link between oral health and teenage quality of life. Fascinatingly, an alignment occurred between human insight and artificial intelligence algorithms in partitioning respondents into distinct groups, unveiling insights that eluded detection through intuitive gender classification alone.

Another notable contribution is the ORIENTATE platform that was presented in a different study [31]. ORIENTATE facilitates the automatic availability of machine learning classification techniques to healthcare professionals, without the need for specialized technical expertise. The platform not only provides a comprehensive report with interpretative graphs but also offers an interface for predicting new input samples. This empowers researchers without specific data technique knowledge to enhance or replace conventional statistical investigations, owing to the feature relevance and interaction plots, which enable precise statistical inference. These innovative approaches signify a transformative leap in dental health assessments, leveraging machine learning and advanced toolkits to bridge existing gaps and enhance overall oral health outcomes.

### 4.8. Supernumerary Tooth Identification

The diagnosis of mesiodens has been conducted using deep learning models [20]. The limited identification of supernumerary teeth on orthopantomographs is primarily attributed to the screening capabilities of less experienced dental personnel [128]. Additionally, a scarcity of proficiency among general dentists exists in recognizing mixed dentition in children. Given these constraints, CNNs have the potential to significantly assist in identifying additional teeth [2]. Researchers [20] detected mesiodens in primary or mixed dentition through AI, and suggested that this approach could expedite and enhance the accuracy of diagnoses for physicians with limited clinical experience. They utilized various deep learning models, including ResNet-18, SqueezeNet, Inception-ResNet-v2, and ResNet-101, based on the premise that deeper networks are more effective in classifying mesiodens.

Significantly, in a study [2] focusing on the detection of supernumerary teeth in the first phase of the transitional dentition stage, three CNN models—AlexNet, VGG16-TL, and Inceptionv3-TL—exhibited outstanding performance. One key strength lies in their open design, facilitating seamless integration into a clinical setting. However, there are notable limitations, including the limited datasets and the capability of AI-driven models to categorize images that are not conducive to two-dimensional panoramic radiography. Enhancing their usage capability in terms of real-world applicability requires the incorporation of a substantial number of medical photos collected from diverse hospitals and institutions into their training.

A study [54] suggested comparing two AI models for 3D segmentation and automatic identification in dental radiography. The additional teeth were identified by Diagnocat Inc. (San Francisco, CA, USA); however, their completely automated program only allows for the export of 3D models. During the automated analysis, the Virtual Patient Creator (Relu, Leuven, Belgium) could not find any extra teeth. This device offers numerous additional functionalities that can be employed for the detection of multiple teeth. Following the initial segmentation, processing methods can be applied to improve the quality of the segmented structures. Enhancements in 3D segmentation techniques aside, it is imperative to use skilled specialists. Because of this, the importance of human knowledge should not diminish, and AI should be seen as an addition to it, rather than a replacement.

Based on the findings of a study, learning methods such as DetectNet and AlexNet exhibited the capability to recognize on 2D orthopantomographs maxillary impacted supernumerary teeth [46]. The study highlighted that this outcome was influenced by the presence of numerous permanent teeth that were still in the process of fully erupting in the patient’s mouth.

Ha et al. [32] demonstrated the effectiveness of their approach in clinical practice for detecting mesiodens in orthopantomographs across all dentition types, in contrast to Kuwada et al.’s study [46], which focused on permanent dentition. The model, based on YOLOv3, exhibited notably superior performance than other DL methods [19].

A study assessed the efficiency of a DL method in identifying permanent tooth germs, suggesting its potential utility in early dental deficiency or extra tooth diagnosis [11]. The study proposed that dentists could save time and effort with access to more precise treatment options. Authors using DL methods [DeepLabV3plus and Inception-ResNet-v2] for mesiodens diagnosis found that a fully automated process was possible, but determining the quantity and position of mesiodens remained challenging [42].

Another study [29] proposed to create and assess an AI model based on a CNN for the diagnosis of taurodontism in teeth using panoramic radiography (PyTorch-implemented U-Net model). The lack of subgroup analysis for maxilla and mandible taurodont teeth was a study drawback, because, in the upper arch, the diagnosis could be limited due to the superimposition of structures (such as the maxillary sinus). The findings of the CNN’s identification of taurodontism were nearly identical to those observed in the designated training dataset, and the system’s ability to identify taurodontism in teeth was practically at the expert level.

The CNN-based deep learning technique shows promise as a diagnostic aid for dentists, although significant advancements in clinical applications are needed before its widespread adoption. The expectation is that a more comprehensive diagnostic system, accommodating various ages and conditions, will be developed in the near future. This advancement could enhance screening for non-pediatric dentists and empower pedodontists to proactively devise treatment strategies.

### 4.9. Early Childhood Caries

A complex disease, influenced by multiple causes in children, is ECC [61,129]. The contributing factors seem unrelated to behavioral and environmental elements, prompting inquiry into the possibility of a biological factor, specifically a hereditary one, playing a more fundamental role in the development of dental caries [78,130]. Researchers have identified numerous genes and gene polymorphisms as potential causes of caries in children. However, the majority of studies have not comprehensively incorporated genetic variables associated with the disease [78,131].

As per Zaorska, K. et al. [78], single-nucleotide polymorphisms (SNPs) could be used to assess the risk of dental caries. This approach could aid in tailoring prevention strategies during a child’s early years and offer guidance to parents in promoting healthier eating habits. The researchers applied artificial neural networks in their study to forecast the likelihood of dental caries based on polymorphisms. Using the information from these forecasts along with early treatment for instances that are discovered might help avoid caries entirely in children and enhance their quality of life in general.

In a recent investigation, researchers compared the effectiveness of ML-based models, including XGBoost, random forest, and LightGBM, with a traditional regression model for early childhood caries detection [61]. The researchers could not detect any significant differences even after using three different machine learning methods, when comparing the results with a logistic regression model. With a restricted feature set, this model can predict preschoolers’ risk of developing ECC by using straightforward tests and questionnaires. Its utility extends to identifying high-risk groups for ECC, implementing proactive preventive interventions, and shaping ECC prevention policies. The main objectives are to reduce the prevalence of early childhood caries and increase the benefits of dental health education for parents of young children.

Another study, conducted by Toledo Reyes et al. [69], evaluated the difference between logistic regression analysis of predictors of primary and permanent teeth caries and an ML approach with 2- and 10-year follow-ups. The 2-year follow-up revealed the models’ highest overall performance, which is to be expected given the behavioral and sociopsychological changes that take place over time. Remarkably, multivariate LR analysis revealed that the only predictor significantly linked to the emergence of new lesions in the primary teeth after two years was the degree of caries at baseline. Additional predictors for dental caries development in primary teeth were found by ML models. These included eating sweet foods frequently and low parental confidence in their child’s oral health. The top-performing models included caries experience in their prediction of caries incidence in permanent teeth, along with a few behavioral and socioeconomic variables (parents’ unemployment, education level, frequency of sugar consumption, and nonuse of fluoride toothpaste). Additionally, ML and LR found that seeing family members less frequently than once a month was a significant predictor. This work suggests that ML might be used in early infancy to predict the development of caries in primary and permanent teeth using easy-to-collect variables.

In their research, Koopaie, M. et al. [44] conducted a comparative analysis of salivary cystatin S levels and demographic data between patients with early childhood caries and those without, employing both statistical analysis and ML techniques. Various learning models, including random forest, XGBoost, feed-forward neural networks, and support vector machines (SVMs), were utilized. The study suggests that salivary cystatin S levels can enhance the effectiveness of ML methods in distinguishing cases of early childhood caries from caries-free controls. Instead of simplifying the identification of crucial components for assessing ECC levels, machine learning approaches enable the creation of computer algorithms capable of considering a multitude of variables and their intricate relationships. This highlights the potential advantages of employing machine learning as a screening tool in the dental sector.

In one study, ref. [37] an automated machine learning application for child classification based on ECC was created and assessed. According to the study’s findings, a parsimonious model performed best in terms of categorization. An ML algorithm can predict the risk of ECC based on the age of the children and the oral health judgments of the parents. Additionally, machine learning can provide highly accurate classifiers that can determine ECC status using demographic and proxy data.

Some authors [59] created a novel caries risk prediction model (CRPM), to consider genetic and environmental factors. Policy-makers may plan the necessary preventative steps for the future by using the CRPM to identify high-risk populations at the community level.

One study [63] examined the potential of screening for dental caries in children by using an ML algorithm to analyze parents’ perceptions of their children’s oral health through a survey. It demonstrates how parent-completed surveys should be taken into consideration to screen for active caries and the experience of caries in children, supporting dental clinicians.

### 4.10. Chronological Age Assessment

Dental age assessment typically employs either the clinical or pantomographic method. While the clinical method is quick but prone to high inaccuracies, the pantomographic approach, focusing on tooth bud mineralization, offers greater precision [76]. Various age assessment methods tailored for different age groups have been developed.

One research study [76] used digital orthopantomography pictures and brain modeling to develop a novel technique for estimating the chronological age of children and adolescents (4–15 years). This approach is easier to use, has almost perfect accuracy, and is notable for being among the first to use pantomographic pictures for metric age determination. One significant drawback is that it only uses OPT images—photos are not included.

Building on Demirjian’s scores, one study [25] utilized ANNs for dental maturity evaluations, revealing that the ages of Malaysian and Indian children and teenagers can be accurately determined using these maturity ratings.

Neural modeling techniques were demonstrated to successfully predict metric age based on proprietary tooth and bone indications in another work [77]. Three deep neural network models were used in the study to determine the chronological age of children and teenagers between the ages of 4 and 15.

An intriguing work by Lee, Y.H. et al. [51] focused mostly on creating machine learning techniques using eighteen radiomorphometric characteristics extracted from orthopantomographs. They observed that ML methods are more effective in estimating age.

A recent research study by Dong et al. [28] evaluated 97 OPT images of 3 to 14-year-old patients in order to forecast each patient’s age based on the phases of seven permanent teeth in the left mandible with SOS-ResNet50, a CNN machine that was originally developed by researchers at Microsoft Research in Washington, DC, USA and applied to the research. Since the third molars are the only permanent teeth that are not fully formed in people between the ages of 14 and 15, the maturity of these teeth is more closely considered when estimating the age of these individuals, leading to a higher mean absolute error among teenagers. The expected dental ages for years 11–15 have been underestimated based on the examination of each age group. Furthermore, the expected dental ages of younger people have been slightly overstated; that is, for those who are 3–7 years old, 8–9 years old, and 10–11 years old. This model has better performance than two already existing methods [70,132].

### 4.11. Detecting Deciduous and Young Permanent Teeth

Various models, including YOLOv3, YOLOv4, Faster R-CNN, and R-CNN, have been employed for object detection and identification. Object detection algorithms can be categorized into two types: the YOLO algorithm, which is a single-stage method, and Mask-R-CNN, R-CNN, and Faster R-CNN, which are two-stage methods.

To identify teeth affected by dental disorders and create links to the detected teeth, automated and sophisticated detection techniques use tooth recognition. CNN-based mapping has demonstrated improved accuracy in automatic tooth segmentation [50]. Digital diagnostic solutions that save time and effort are getting closer to reality in dentistry with the use of panoramic radiography for numbering primary or permanent teeth [40].

The article conducted by Dong et al. [28] used the YOLOv3 algorithm to identify each tooth in an OPG image and give it the correct tooth number. Using YOLOv4 [38], an object identification model based on CNNs, researchers evaluated the performance of a DL method for automatic tooth detection and counting. The model displayed proficiency in identifying and enumerating both permanent and primary teeth. The outstanding speed and accuracy of YOLOv4 established it as the favored choice for object detection.

Caliskan, S. et al. [26] used CNN algorithms to identify and classify unerupted molars, and they discovered that this method worked well. To find out if a specific tooth germ is missing using teeth-numbering algorithms, more study is required. Finding missing tooth germs may help dentists create more individualized treatment regimens.

A speedier R-CNN Inceptionv2 technique was examined by some authors [40] to identify and count deciduous teeth in children’s orthopantomographs. The approach identified and numbered only deciduous teeth with remarkable sensitivity and accuracy ratings. Considering the importance of primary teeth in forensic identification, this distinction is remarkable.

In order to identify and number primary teeth on pediatric panoramic radiographs, Kilic, M.C. et al. looked at a quicker R-CNN Inceptionv2 methodology. They found that the method had good sensitivity and accuracy scores. Only primary teeth were found and numbered, which is noteworthy because primary teeth are crucial for forensic identification.

Another R-CNN method was proposed [24] to identify teeth, both deciduous and permanent ones, in OPT images, and their eventually associated fillings. This study obtained high accuracy-level results, and dentists can use this information to enhance their radiograph interpretations, help convey the information to patients, and support dental students learning to read radiographs by creating ML models to evaluate orthopantomographs.

In structuring the discussion, it is important to highlight that, despite their often superior accuracy, two-stage detectors demand more time and processing power compared to one-stage ones. Conversely, YOLO serves as an exemplar of a single-stage detector adept at rapidly and accurately classifying items. Moreover, instead of other CNN models, it has a superior real-time object recognition and a consistent above-average performance across a diverse array of object classes.

### 4.12. Application of AI in Adult and Pediatric Dentistry

Because of the unique requirements and difficulties that each environment presents, the applications of artificial intelligence (AI) in adult and pediatric dentistry differ greatly.

In adult dentistry, artificial intelligence (AI) is used to analyze radiographic pictures, CT scans, and clinical data to improve the diagnosis and treatment of dental disorders. AI systems are able to spot patterns and abnormalities that the human eye might miss, which makes it easier for medical professionals to spot pathologies like gum disease, caries, and oral lesions early on. Because of this, diagnoses may be made more quickly and accurately, lowering the possibility of mistakes and increasing treatment efficacy overall.

Furthermore, by simplifying workflow management, treatment planning, and patient communication, AI can improve the operational effectiveness of dental offices. AI-powered patient management systems can schedule appointments automatically, notify patients when they have appointments, and make it easier for medical professionals to share information.

Conversely, artificial intelligence in pediatric dentistry is primarily concerned with behavior management and oral health education for younger children. Interactive games and instructional apps are created to keep kids interested and support the formation of good hygiene habits at a young age. To make learning enjoyable and interesting, these systems could make use of gamification and incentive strategies.

Additionally, AI in pediatric dentistry can be utilized to tailor care to each child’s unique needs. AI algorithms, for instance, can provide therapeutic strategies that are more suited for kids with specific sensitivities or dental issues, or they can assist in determining the most ideal anesthesia dosage based on the patient’s age and weight.

AI aims to enhance the overall patient experience in both situations by lowering dental care-related anxiety and discomfort and encouraging improved long-term oral health. The particular uses, however, vary according to the features of the patients and the therapeutic difficulties that range across various demographic categories. Table 3 shows the possible field of application of AI in pedodontics.

### 4.13. Strengths and Limitations

This literature review focused on the different fields of AI in pedodontics and analyzed all current studies published on this topic. The high number of cited articles reflects the number of studies evaluated. Additionally, it is worth acknowledging that the number of publications addressing the utilization of AI in dentistry, specifically in pedodontics, is steadily increasing each year. Consequently, there was a requirement for an updated review extending until December 2023.

The lack of global and organized standards for AI development was identified as the main barrier to its efficacy. Furthermore, difficulties with gathering comprehensive data, guaranteeing accessibility, upholding appropriate data structures, and accomplishing comprehensiveness were noted [3]. However, additional queries are coming up concerning the morality, obligations, value, and applications of AI in human life. The main reasons for dissatisfaction among dental practitioners were the challenges of protecting patients’ privacy and their unwillingness to adopt AI-based techniques, given the need to preserve human interaction in clinical care.

Nevertheless, considering the efficacy of these AI models, it is imperative to create and implement guidelines to have these models promoted and used in clinical settings as soon as possible. This will help medical practitioners diagnose patients and choose the best course of action.

### 4.14. Future Applications of AI

AI in pediatric dentistry has the potential to completely transform the field by improving patient care, shortening procedures, and encouraging youngsters to have healthier teeth. The following are some possible areas for growth:

- AI-assisted diagnosis systems: Pediatric dentists should be able to diagnose cavities, dental abnormalities, and other oral pathologies in children early on with the use of sophisticated AI algorithms. To detect indications of dental problems swiftly and precisely, these systems might make use of natural language processing, radiography image analysis, and other clinical data.
- Personalization of treatment: AI might be used to tailor treatment regimens to the unique requirements of every child. This could entail figuring out the ideal treatment plan for a given dental disease while taking the patient’s age, general health, and preferences into account.
- Virtual assistance and tele-dentistry: AI-driven systems may be created to facilitate remote dental consultations and pediatric patient telemonitoring. These options may be especially helpful for families that do not have easy access to dental care or live in distant places.
- Automated learning and patient education: AI technologies have the potential to create engaging, customized educational programs for children that teach them good hygiene practices, make dental treatments understandable, and motivate them to take an active role in their oral healthcare.
- Patient behavior management: AI may be used to create sophisticated algorithms that control how young patients behave when they see the dentist. These systems could provide children with a cozy and comforting environment throughout therapies by utilizing interactive technologies and child psychology-based approaches.

AI may also enable predictive analytics to use patient data to foresee possible problems with oral health, enabling proactive interventions and preventative actions. Furthermore, dental professionals may be able to detect patterns and trends in patient populations with the aid of AI-driven data analytics, which could result in more successful public health campaigns and legislation to support pediatric oral health.

In conclusion, the application of AI to pediatric dentistry has the potential to enhance treatment effectiveness, foster better communication between patients and dental professionals, and lessen patient anxiety about dental appointments—all of which can help support children’s oral health and general well-being. Further research and applications of AI-driven solutions in pediatric dentistry offer the potential for revolutionizing the sector and improving the standard of care given to young patients as technology continues to progress.

## 5. Conclusions

The early applications of AI in pedodontics focused on analyzing radiographic images and providing diagnostic support. AI systems were trained to automatically identify cavities and dental and skeletal anomalies, facilitating dentists’ work and accelerating diagnosis times. Another significant step was the development of intelligent medical records. These digital systems integrate the patient’s health data, allowing a complete and organized view of their medical history. AI can analyze these data to identify potential oral health problems, suggest preventive and personalized treatments, and alert the dentist to any risk factors.

Moreover, because it provides trustworthy and efficient solutions across several industries, AI is growing in popularity in the field of pedodontics, as reported in the present review. Subsequent efforts may concentrate on the creation of cloud-based frameworks intended to streamline data integration and encourage cooperative data exchange. Utilizing vast volumes of high-quality data can enhance the accuracy of prediction results and picture interpretation when employing ML techniques, since data are the fundamental building elements of robust models. An AI model that has been properly trained may be able to assist in the screening and diagnosis of growing patients in the field of pedodontics research.

## Figures and Tables

**Table 1:** Explanation of AI terminology.

AI Term	Explanation
Artificial intelligence (AI)	The primary objective of artificial intelligence (AI) is to build intelligent computers that can learn from data and find solutions on their own. When machines are given new information, they may utilize statistical and probabilistic approaches to learn from past examples and make better decisions. Learning, the process by which behavior or performance is enhanced via practice and experience, is a necessary component of intelligent systems [5]. AI is being used in medicine to replace the manual standards that were previously used in machine learning (ML) and deep learning (DL) [6].
Machine learning (ML)	Machine learning (ML) uses computers to build statistical models and algorithms that improve understanding and reasoning [7]. It entails training algorithms on massive datasets to identify patterns, which are subsequently applied to forecast or choose fresh data [8].
Deep learning (DL)	Deep learning (DL) is a subfield of machine learning that uses artificial neural networks to mimic how the human brain learns [9]. Because they are trained using a large amount of data and algorithms, they are more accurate [6]. Layers of tiny communication units called neurons make up an artificial neural network (ANN), a kind of deep learning structure. An ANN with several hidden layers is all that deep learning is. A subset of ANNs called convolutional neural networks (CNNs) are mostly used in general medicine and dentistry [7,10]. CNNs, a subclass of DL, use fully connected layers plus a subsampling layer that resembles a multilayer perceptron to analyze an image with visual cortex cells [11].
Big data	The term “big data” refers to large datasets and/or the compilation of all accessible data from many sources. It may be utilized to identify patterns that result in unique experiences for different individuals [12].

**Table 2:** Articles included in the review.

Author/Year/Country/Journal	Sample	AI Method	Type of Study	Results	Objective
Ahn 2021 [20], Korea, *Diagnostics*	1100 patients	SqueezeNet, ResNet-18, ResNet-101, and Inception-ResNet-v2	Retrospective study	ResNet-101 and Inception-ResNet-v2 both have scores, recall, accuracy, and precision greater than 90%. SqueezeNet produced outcomes that were comparatively worse.	The objective of this research was to create and assess deep learning models that automatically identify mesiodens in panoramic radiographs with primary or mixed dentition.
Amasya 2020 [21], Turkey, *American Journal of Orthodontics and Dentofacial Orthopedics*	647 patients	ANN model	Retrospective study	The ranges of intraobserver agreement were wκ = 0.92–0.98, cκ = 0.65–0.85, and 70.8–87.5%. The ranges of interobserver agreement were wκ = 0.76–0.92, cκ = 0.45–0.65, and 50–72.2%. Observers 1, 2, 3, and 4 and the ANN model agreed on the following values: wκ = 0.85 (cκ = 0.52, 59.7%), wκ = 0.8 (cκ = 0.4, 50%), wκ = 0.87 (cκ = 0.55, 62.5%), and wκ = 0.91 (cκ = 0.53, 61.1%), respectively (*p* < 0.001). The ANN model and the human observers showed an average agreement of 58.3%.	The objective of this study was to create an artificial neural network (ANN) model for the analysis of cervical vertebral maturation (CVM) and compare the model’s output to observations made by human observers.
Arik 2017 [22], USA, *Journal of Medical Imaging (Bellingham)*	250 patients	CNN	Retrospective study	The findings show good anatomical type classification accuracy (~76% average classification accuracy for test set) and excellent anatomical landmark detection accuracy (~1% to 2% greater success detection rate for a 2 mm range compared with the top benchmarks in the literature).	The aim of this study was to use deep convolutional neural networks (CNNs) for completely automated quantitative cephalometry.
Bag 2023 [23], Turkey, *BMC Oral Health*	981 patients	YOLOv5	Retrospective study	A total of 14,804 labels were created, including those for the mandibular canal (1879), maxillary sinus (1922), orbit (1944), mental foramen (884), foramen mandible (1885), incisura mandible (1922), articular eminence (1645), and condylar (1733) and coronoid (990) processes. The orbit (1), incisura mandible (0.99), maxillary sinus (0.98), and mandibular canal (0.97) yielded the highest F1 scores. The orbit, maxillary sinus, mandibular canal, incisura mandible, and condylar process yielded the best sensitivity levels. The articular eminence (0.92) and mental foramen (0.92) had the lowest sensitivity ratings.	The aim of the research was to examine the efficacy and dependability of artificial intelligence in identifying maxillary and mandibular anatomic components shown on panoramic radiographs in children.
Bumann 2023 [24], USA, *Journal of Dentistry*	448 patients	CNN	Retrospective study	Models that can distinguish between primary and permanent teeth (mean average precision [mAP] 95.32% and performance [F-1] 92.50%), as well as the dental fillings that are connected to them (mAP 91.53% and F-1 91.00%) were created by using these high-performance classifiers. Additionally, a brand-new approach to cooperative learning was created that makes use of these two classifiers to improve recognition performance (mAP 94.09 percent and F-1 93.41 percent).	The objective of this work was to create a novel collaborative learning model that simultaneously identified and distinguished between primary and permanent teeth as well as detected fillings in order to support the assessment of panoramic radiographs.
Bunyarit 2021 [25], Malaysia, *Pediatric Dental Journal*	1569 patients	ANN model	Retrospective study	An initial comparison of estimated DA based on dental maturity scores from Chaillet and Demirjian with known CA revealed that DA was consistently overestimated for all age groups, with 2.09 ± 0.90 years for Malay boys and 2.79 ± 0.99 years for Malay girls (paired *t*-test, *p* < 0.05). An adaption utilizing artificial neural networks was used to create new dental maturity scores (NDA) that were more appropriate for the Malay individuals in order to improve the age estimation. According to the paired *t*-test results (*p* > 0.05), there was a greater degree of accuracy in determining dental age (0.035 ± 0.84 years for boys and 0.048 ± 0.928 years for girls).	The purpose of this study was to evaluate the applicability of the Chaillet and Demirjian-introduced 8-tooth approach on Malaysian children aged 5.00–17.99 years and to produce teeth maturity ratings for age estimation based on artificial neural networks (ANNs) that are more accurate.
Caliskan 2021 [26], Turkey, *International Journal of Computerized Dentistry*		CNN	Retrospective study	When compared to the reference standard, the system’s identification and categorization had a high success rate. When compared to observers, the system’s performance was incredibly accurate.	Comparing the effectiveness and dependability of an artificial intelligence (AI) program for the identification and categorization of submerged teeth in panoramic radiographs was the study’s main goal.
Chen 2020 [27], China, *Angle Orthodontist*	60 patients	LINKS	Prospective study	With an average dice ratio of 0.80 for three-dimensional image segmentations and a minimal mean difference of two voxels on the midsagittal plane for digitized landmarks between the manually identified and the machine learning-based (LINKS) methods, the maxillary structure was successfully auto-segmented. Between the impaction ([2.37 ± 0.34] × 10^−4^ mm^3^) and nonimpaction ([2.36 ± 0.35] × 10^−4^ mm^3^) sides of SG, there was no discernible variation in bone volume. Comparing the SG and CG maxillae, the former exhibited considerably lower volumes, widths, heights, and depths (*p* < 0.05).	In an effort to advance therapeutically useful knowledge, the aim of the research was to (1) provide a novel machine learning technique; and (2) evaluate maxillary structural variation in unilateral canine impaction.
Dong 2023 [28], China, *Applied Intelligence*		YOLOv3 and SOS-Net	Prospective study	With an F1 score improvement of 3.77%, the ablation trials and visual analysis of SOS-Net confirmed the efficacy and interpretability of the SFC block and ASA loss. Furthermore, comparison trials show that the suggested methodology outperforms cutting-edge techniques in maturity staging and age estimation.	In this study, an automatic system for determining the whole permanent dentition’s tooth maturity phases was developed.
Duman 2023 [29], Turkey, *Oral Radiology*	434 patients	CNN	Retrospective study	There were 29 false positives, 17 false negatives, and 109 genuine positives out of the 43 test group photos with 126 labels. The results of the taurodont tooth segmentation process were 0.8650, 0.7898, and 0.8257 for sensitivity, precision, and F1 score, respectively.	The goal of the work was to create and assess an AI model based on a CNN for the diagnosis of taurodontism in teeth using panoramic radiography.
Felsch 2023 [30], Germany, *NPJ Digital Medicine*	18,179 images	SegFormer-B5	Retrospective study	For the fine-tuned model, the overall diagnostic performance was 0.959, 0.977, and 0.978 in terms of IoU, AP, and ACC. The most significant caries classes of dentin cavities (0.692, 0.830, and 0.997) and non-cavitations (0.630, 0.813, and 0.990) had high levels of matching data. Similar outcomes were seen for atypical restoration (0.829, 0.902, and 0.999) for delimited opacity associated with MIH (0.672, 0.827, and 0.993).	The goal of this work was to create an AI-based algorithm that can identify, categorize, and pinpoint MIH and caries.
Gajic 2021 [8], Serbia, *Children*	374 patients	CNN	Retrospective study	Both human intuition and computer algorithms arrived at the same result on the optimal division of respondents. Thus, it was shown that the technique has high quality and that there is a necessity of carrying out these kinds of analyses in dental research.	This study aimed to assess the effects of dental health on adolescents’ quality of life using both artificial intelligence algorithms and conventional statistical approaches.
Gomez-Rios 2023 [31], Spain, *BMC Oral Health*	274 patients	ORIENTATE	Case study	As a case study, its application to a dataset of children with special healthcare requirements (SHCN) who were healthy and receiving deep sedation was presented. The feature selection technique on the sample dataset, in spite of its tiny size, identified a collection of characteristics with an F1 score of 0.83 and an ROC (AUC) of 0.92 that might predict the need for a second sedation. Eight predictive factors were identified and ranked according to the model’s significance for each of the two groups. A comparison with a traditional study and a discussion that drew conclusions from the relevance and interaction plots were also given.	The aim of the work was to present and explain ORIENTATE, a software that enables clinical practitioners without specialized technical knowledge to automatically use machine learning classification methods.
Ha 2021 [32], Korea, *Scientific Reports*	612 patients	YOLOv3	Retrospective study	In the original photos, the accuracy of the external test dataset was 89.8%, while the accuracy of the internal test dataset was 96.2%. The accuracies of the exterior test dataset were 86.7%, 95.3%, and 86.7% for the primary, mixed, and permanent dentition, respectively, whereas the accuracies of the internal test dataset were 96.7%, 97.5%, and 93.3%. In both test datasets, the CLAHE images produced less accurate findings than the original photos.	The goal of this work was to create an AI model that can identify mesiodens on panoramic radiographs of different dentition groups.
Hansa 2021 [33], South Africa, *American Journal of Orthodontics and Dentofacial Orthopedics*	90 patients	Dental Monitoring	Prospective study	In terms of sample size, age, gender, angle categorization, maxillary and mandibular irregularity index, and the number of initial aligners, the two groups were homogeneous (*p* > 0.05). In the DM group, there was a substantial (*p* = 0.001) drop in the number of appointments, by 3.5 visits (33.1%). The DM group also experienced a significant (*p* = 0.001) decrease in the time to the first refinement (1.7 months). Final tooth positions were statistically (*p* < 0.05) more accurate for the DM group in comparison to the Invisalign-anticipated positions for the maxillary anterior dentition in rotational motions and the mandibular anterior dentition in buccal–lingual linear movement. For the maxillary posterior teeth, Invisalign therapy without DM was more in line with anticipated tooth placements at the tip. The clinically relevant criteria (>0.5 mm or >2°) were not exceeded by any of these variations, yet the DM group did so in 1.7 fewer months.	The results of Invisalign clear aligner therapy were compared for treatment length, number of sessions, refinements and refinement aligners, and Invisalign’s accuracy in achieving projected tooth positions (aligner tracking) with and without Dental Monitoring (DM).
Hansa 2021 [34], South Africa, *Seminars in Orthodontics*		Artificial intelligence-driven remote monitoring	Case series	A few advantages that AI-driven remote monitoring (AIRM) provides for patient care before, during, and after treatment for aligners and fixed appliances are illustrated graphically in this clinical display. Surprisingly, remote monitoring actually helps with patient–physician communication. This allows the clinician to keep a closer eye on the patient and speak with them directly as needed.	Reviewing the clinical applicability and justification of orthodontic remote monitoring was the goal of this case series.
Hwang 2020 [35], Korea, *Angle Orthodontist*	1028 cephalograms	YOLOv3	Retrospective study	In contrast to the human intraexaminer variability of repeated manual detections, which showed a detection error of 0.97 ± 1.03 mm, artificial intelligence consistently detected similar positions on each landmark after multiple trials. Between humans and AI, the mean detection error was 1.46 ± 2.97 mm. Between human examiners, the mean difference was 1.50 ± 1.48 mm. Less than 0.9 mm separated the detection errors made by AI and human examiners on average, and this difference did not appear to be clinically significant.	The aim of the study was to compare the detection patterns of 80 cephalometric landmarks found by an automated identification system (AI) with those found by human examiners. The AI was based on a recently suggested deep learning technology called You-Only-Look-Once version 3 (YOLOv3).
Iglovikov 2018 [36], USA, *bioRxiv*	12,600 images	CNN	Retrospective study	The accuracy was higher in region C, which is composed of the proximal phalanges and metacarpals, with an MAE of 8.42 months. The MAE for region A (the entire image) was 8.08 months. With an MAE of 7.52 months, the linear ensemble of the three regional models performed better than other models mentioned in the study. With very few exceptions, the geographical trend MAE(B) > MAE(C) > MAE(A) > MAE (ensemble) was also shown for various model types and patient cohorts.	In this research, data from the 2017 Pediatric Bone Age Challenge, hosted by the Radiological Society of North America, were used to demonstrate the applicability of a fully automated deep learning method to the problem of bone age assessment.
Karhade 2021 [37], USA, *Pediatric Dentistry*	6404 patients	AutoML	Retrospective study	The highest AUC (0.74), Se (0.67), and PPV (0.64) scores were obtained by a parsimonious model that included two terms (i.e., children’s age and parent-reported child oral health status: excellent/very good/good/fair/poor). This model also performed similarly when an external National Health and Nutrition Examination Survey (NHANES) dataset was used (AUC equaled 0.80, Se equaled 0.73, and PPV equaled 0.49). On the other hand, a comprehensive model with 12 factors that included dental home, fluoride exposure, oral health behaviors, parental education, and race/ethnicity performed worse (AUC equaled 0.66, Se equaled 0.54, and PPV equaled 0.61).	The aim of the research was to design and assess an automated machine learning algorithm (AutoML) for the classification of children based on the status of early childhood caries (ECC).
Kaya 2022 [11], Turkey, *Imaging Science in Dentistry*	4518 patients	YOLOv4	Retrospective study	With an average precision value of 94.16% and an F1 value of 0.90, the YOLOv4 model, which identified permanent tooth germs on pediatric panoramic radiographs, demonstrated a high degree of relevance. 90 ms was the average YOLOv4 inference time.	This study evaluated a deep learning system’s efficacy in detecting permanent tooth germs on pediatric panoramic radiographs.
Kaya 2022 [38], USA, *Journal of Clinical and Pediatric Dentistry*	4545 patients	YOLOv4	Retrospective study	Using pediatric panoramic radiographs, the model successfully identified and numbered both primary and permanent teeth, with a weighted F1 score of 0.91, a mean average precision (mAP) value of 92.22%, and a mean average recall (mAR) value of 94.44%. When it came to automated tooth detection and numbering on pediatric panoramic radiographs, the suggested CNN approach performed well and quickly.	The goal of this paper was to assess how well a deep learning system performed on pediatric panoramic radiographs for automated tooth recognition and numbering.
Kaya 2023 [39], USA, *International Journal of Computerized Dentistry*	4821 patients	YOLOv4	Retrospective study	With high F1 values of 0.95, 0.90, and 0.76 for immature teeth, permanent tooth germs, and brackets, respectively, the YOLOv4 model accurately diagnosed these conditions. Despite the encouraging outcomes, this model has certain drawbacks for a few dental procedures and structures, such as fillings, root canals, and extra teeth.	This study set out to assess how well a deep learning software system performed in identifying and categorizing dental procedures and structures on pediatric patients’ panoramic radiographs.
Kilic 2021 [40], Turkey, *Dentomaxillofacial Radiology*	421 images	CNN	Retrospective study	The artificial intelligence system was able to identify and classify children’s deciduous teeth based on panoramic radiographs. There were high rates of precision and sensitivity. According to estimates, the F1 score, precision, and sensitivity were 0.9686, 0.9571, and 0.9804, respectively.	This study assessed the automated identification and tagging of children’s deciduous teeth as seen on panoramic radiographs using a deep learning technique.
Kim 2021 [41], Korea, *Orthodontics and Craniofacial Research*	455 patients	CNN	Retrospective study	Eight machine learning models made up the final ensemble model. The RMSE, round MAE, and MAE were, in that order, 1.20, 0.87, and 0.90.	The aim of this study was to use cervical vertebrae (CV) pictures to forecast the stages of hand–wrist maturation and evaluate the precision of the suggested methods.
Kim 2022 [42], Korea, *Dentomaxillofacial Radiology*	988 radiographs	DeepLabV3plus and Inception-ResNet-v2	Comparative study	The mean BF score was 0.907, the IoU was 0.762, and the segmentation performance utilizing posterior molar space in panoramic radiographs was 0.839. For the diagnosis of mesiodens using automatic segmentation, the mean values of accuracy, precision, recall, F1 score, and area under the curve were, respectively, 0.971, 0.971, 0.971, and 0.971.	The goal of this research was to create and assess the efficacy of a deep learning model that uses an automatic region of interest (ROI) setting and mesiodens diagnosis in panoramic radiographs of developing youngsters.
Kok 2019 [43], Turkey, *Progress in Orthodontics*	300 patients	k-Nearest neighbors (k-NN), naive Bayes (NB), decision tree (Tree), artificial neural network (ANN), support vector machine (SVM), random forest (RF), and logistic regression (Log.Regr.) algorithms.	Comparative study	The methods with the highest accuracy in detecting cervical vertebrae stages were k-NN: CVS 5 (60.9%)–CVS 6 (78.7%) and SVM: CVS 3 (73.2%)–CVS 4 (58.5%) and CVS 1 (97.1%)–CVS 2 (90.5%), according to a confusion matrices decision tree. With the exception of CVS 5, which had the third-highest accuracy value of 47.4%, the ANN algorithm was found to have the second-highest accuracy values (93%, 89.7%, 68.8%, 55.6%, and 78%, respectively) in identifying all stages. The most stable algorithm was the ANN, with an average rank of 2.17, based on how well the algorithms predicted the CVS classes.	The goal of this research was to compare the performance of these algorithms and identify the cervical vertebrae stages (CVSs) for growth and development periods using the seven most popular artificial intelligence classifiers.
Koopaie 2021 [44], Iran, *BMC Oral Health*	40 patients	Human cystatin S ELISA kit	Comparative study	In the early childhood caries group, the mean salivary cystatin S concentration was 191.55 ± 81.90 (ng/mL), while in the caries-free group, it was 370.06 ± 128.87 (ng/mL). Salivary cystatin S levels in early childhood caries and caries-free groups differed statistically significantly (*p* = 0.032), according to a *t*-test study. The logistic regression model based on salivary cystatin S levels and birth weight showed the highest, and an acceptable, potential for differentiating early childhood caries from caries-free controls, according to an analysis of the area under the curve (AUC) and accuracy of the ROC curve. Moreover, data about salivary cystatin S levels improved the machine learning techniques’ capacity to distinguish early childhood caries from controls without caries.	This work used statistical analysis and machine learning techniques to evaluate salivary cystatin S levels and demographic information in early childhood caries cases compared to caries-free cases.
Kunz 2020 [45], Germany, *Journal of Orofacial Orthopedics*	50 patients	CNN	Comparative study	The AI’s forecasts and the gold standard set by humans nearly never differed statistically. It appears that there are no clinically significant differences between the two analyses.	The purpose of this study was to develop an artificial intelligence (AI) program specifically designed to generate an automated cephalometric X-ray examination.
Kuwada 2020 [46], Japan, *Oral Surgery, Oral Medicine, Oral Pathology and Oral Radiology*	550 patients	AlexNet, VGG16, and DetectNet	Comparative study	In general, DetectNet yielded the greatest diagnostic effectiveness results. By comparison, VGG16 produced substantially lower values than DetectNet and AlexNet. Recall, precision, and the F-measure for detection in the incisor region were all 1.0, indicating faultless detection, according to an evaluation of DetectNet’s detection capability.	The purpose of this study was to evaluate and compare the efficacy of three deep learning algorithms for the classification of individuals with fully erupted incisors who have maxillary impacted supernumerary teeth (ISTs).
Larson 2018 [47], USA, *Radiology*	200 images	CNN	Comparative study	With a mean RMS and MAD of 0.63 and 0.50 years, respectively, the mean difference between the reviewers’ and the model’s predictions of bone age was zero years. The three reviewers’ estimates, the clinical report’s estimate, and the model’s estimate all fell within a 95% confidence interval. The RMS for the Digital Hand Atlas dataset was 0.73 years, as opposed to a previously published model’s result of 0.61 years.	The aim of this research was to examine how well an automated deep learning bone age assessment model that uses hand radiographs performed in comparison to existing automated models and experienced radiologists.
Lee 2017 [48], USA, *Journal of Digital Imaging*	1200 images	ImageNet	Retrospective study	Using held-out test photos, the models obtained 57.32 and 61.40% accuracies for the female and male cohorts, respectively, using an ImageNet pretrained and fine-tuned convolutional neural network (CNN). In 1 year, 90.39% of female test radiographs had a BAA assigned, and in 2 years, 98.11% of them did. In one year, 94.18% of male test radiographs were allocated, and in two years, 99.00%. The attention maps that showed the features the trained model utilized for BAA were made using the input occlusion approach.	This paper presented a fully automated deep learning pipeline designed to run BAA, normalize and preprocess input radiographs, and segment a region of interest.
Lee 2020 [49], Korea, *BMC Oral Health*	400 patients	Bayesian Convolutional Neural Networks (BCNNs)	Retrospective study	In the 2, 3, and 4 mm range, the framework demonstrated a mean landmark error (LE) of 1.53 ± 1.74 mm and attained successful detection rates (SDRs) of 82.11, 92.28, and 95.95%, respectively. In particular, the gonion, the most inaccurate location in earlier research, almost halved its error when compared to the other points. Furthermore, these findings showed a noticeably improved ability to recognize anatomical anomalies. The framework can help with clinical convenience and decision-making by offering confidence regions (95%) that take uncertainty into account.	In this work, the authors sought to create a novel framework that makes use of Bayesian Convolutional Neural Networks (BCNNs) to locate cephalometric landmarks with confidence areas.
Lee 2020 [50], Korea, *Oral Surgery, Oral Medicine, Oral Pathology and Oral Radiology*	50 radiographs	CNN	Prospective study	The suggested approach yielded a mean IoU of 0.877 and an F1 score of 0.875 (precision: 0.858, recall: 0.893). The segmentation method’s visual inspection revealed a strong similarity to the actual data.	The aim of this paper was to assess a fully deep learning mask region-based convolutional neural network (R-CNN) technique that uses individual annotation of panoramic radiographs for automatic teeth segmentation.
Lee 2022 [51], Korea, *Scientific Reports*	471 patients	CNN	Prospective study	The AUCs achieved for classifying young ages (10–19 years old) in the prediction of three age-group categories ranged from 0.85 to 0.88 for five different machine learning models. The AUC value for adult individuals (20–49 years old) was roughly 0.73, whereas those for the elder age group (50–69 years old) varied from 0.82 to 0.88. With mean AUCs ranging from 0.85 to 0.87 and 80 to 0.90, respectively, age groups 1 (10–19 years old) and 6 (60–69 years old) had the best scores among the six age-group classifications. Based on LDA weights, a feature analysis revealed that the L-Pulp Area was significant in differentiating between young people (10–49 years old), while L-Crown, U-Crown, L-Implant, U-Implant, and periodontitis served as predictors for elderly people (50–69 years).	The objective of this research was to examine the correlation between 18 radiomorphometric characteristics of panoramic radiographs and age, and to employ five machine learning algorithms to accurately and non-invasively predict the age group of individuals with permanent teeth.
Lo Giudice 2021 [52], Italy, *Orthodontics and Craniofacial Research*	40 patients	CNN	Retrospective study	The ICC score of 0.937 indicated a strong correlation between the measurements, and there was a technique error of 0.24 mm^3^. There was a 0.71 (±0.49) cm^3^ difference between the approaches, although *p* > 0.05 indicated that the difference was not statistically significant. Results of 90.35% (±1.88) (tolerance 0.5 mm) and 96.32% (±1.97%) (tolerance 1.0 mm) matched percentages were found. The DSC percentage discrepancies in the assessments conducted using the two methodologies were 2.8% and 3.1%, respectively.	The aim of this paper was to assess the efficacy of a fully automated deep learning-based technique for mandibular segmentation from CBCT images.
Mahto 2022 [53], Nepal, *BMC Oral Health*	30 patients	WebCeph	Comparative study	The ICC value for each measurement was more than 0.75. Seven parameters (ANB, FMA, IMPA/L1 to MP (°), LL to E-line, L1 to NB (mm), L1 to NB (°), S-N to Go-Gn) had ICC values greater than 0.9, while five parameters (UL to E-line, U1 to NA (mm), SNA, SNB, and U1 to NA (°)) had ICC values between 0.75 and 0.90.	The aim of this study was to assess the validity and reliability of automated cephalometric measurements obtained from “WebCeph”^TM^ and to compare the linear and angular cephalometric measurements obtained from the web-based fully automated artificial intelligence (AI)-driven platform with those from manual tracing.
Mine 2022 [2], Japan, *International Journal of Pediatric Dentistry*	220 radiographs	AlexNet, VGG16-TL, and Inceptionv3-TL	Comparative study	In terms of accuracy, sensitivity, specificity, and area under the ROC curve, the VGG16-TL model performed best; nevertheless, the other models displayed comparable performance.	The purpose of this study was to use deep learning based on convolutional neural networks (CNNs) to identify children in the early stages of mixed dentition who had excess teeth.
Mladenovic 2023 [54], Serbia, *Diagnostics*	1 patient	CNN	Case study	Multiple tools must be used in practice because there is currently no one tool that can meet all clinical needs regarding supernumerary teeth and their segmentation.	The aim of this study was to examine the possibilities, advantages, and drawbacks of artificial intelligence; the authors offered a highly rare instance of a youngster with nine extra teeth together with two commercial tooth segmentation technologies.
Mohammad-Rahimi 2022 [55], Iran, *Korean Journal of Orthodontics*	890 radiographs	CNN	Retrospective study	For the six-class CVM diagnosis, the model’s validation and test accuracies were 62.63% and 61.62%, respectively. Furthermore, the three-class classification’s validation and test accuracies for the model were 75.76% and 82.83%, respectively. Moreover, significant agreements were noted between one of the two orthodontists and the AI model.	By examining lateral cephalometric radiographs, a novel deep learning model for assessing growth spurts and cervical vertebral maturation (CVM) degree was presented and evaluated in this work.
Montufar 2018 [56], Mexico, *American Journal of Orthodontics and Dentofacial Orthopedics*	24 patients	CNN	Retrospective study	This method produced a 3.64-mm mean error in the localization of cephalometric landmarks from the 3D gold standard. The porion and sella regions had the highest localization errors due to their limited volume definition.	Using an active shape model to look for landmarks in related projections, this paper proposed a novel method for automatic cephalometric landmark localization on three-dimensional (3D) cone beam computed tomography (CBCT) volumes.
Niño-Sandoval 2016 [57], Colombia, *Forensic Science International*	229 radiographs	Support vector machine	Retrospective study	The angles Pr-A-N, N-Pr-A, A-N-Pr, A-Te-Pr, A-Pr-Rhi, Rhi-A-Pr, Pr-A-Te, Te-Pr-A, Zm-A-Pr, and PNS-A-Pr were used to define accuracy, which came out to be 74.51%. Class II and Class III were correctly distinguished from one another, as demonstrated by the class precision and class recall.	The purpose of this work was to recreate the normal mandibular position on a modern Colombian sample by using an automatic non-parametric technology called support vector machines to classify skeletal patterns using craniomaxillary factors.
Nishimoto 2019 [58], Japan, *Journal of Craniofacial Surgery*	219 images	CNN	Prospective study	Prediction errors were 17.02 pixels on average and 16.22 pixels on the median. In cephalometric analysis, the neural network’s predicted angles and lengths did not differ statistically from those derived from manually plotted points.	The authors developed a deep learning neural network-based automatic landmark prediction system.
Pang 2021 [59], China, *Frontiers in Genetics*	1055 patients	CNN	Prospective study	Cohort 2 (the testing cohort) had an AUC of 0.73 for the caries risk prediction model, demonstrating a good degree of discrimination ability. The caries risk prediction algorithm was able to correctly identify people with high and very high caries risk, but it underestimated the risks for people with low and very low caries risk, according to risk stratification.	The purpose of this work was to use a machine learning algorithm to build a new model for predicting teenage caries risk based on genetic and environmental factors.
Park 2019 [60], Korea, *Angle Orthodontist*	283 images	You-Only-Look-Once version 3 (YOLOv3) and Single Shot MultiBox Detector (SSD) methods.	Retrospective study	For 38 out of 80 landmarks, the YOLOv3 algorithm performed more accurately than the SSD. There was no statistically significant difference between YOLOv3 and SSD for the remaining 42 out of 80 markers. YOLOv3 error plots revealed a more isotropic trend in addition to a reduced error range. For YOLOv3 and SSD, the average computational times per image were 2.89 s and 0.05 s, respectively. YOLOv3 had an accuracy that was around 5% greater than the best standards reported in the literature.	The aim of this paper was to evaluate two of the most recent deep learning algorithms for the automatic recognition of cephalometric landmarks in terms of accuracy and computational efficiency.
Park 2021 [61], Korea, *International Journal of Environmental Research and Public Health*	4195 patients	XGBoost (version 1.3.1), random forest, and LightGBM (version 3.1.1)	Comparative study	When the misclassification rates and area under the receiver operating characteristic (AUROC) values of the various models were evaluated, it was found that the AUROC values of the four prediction models ranged from 0.774 to 0.785. Moreover, there was no discernible variation found in the AUROC values of the four models.	In this study, the objective was to create prediction models for early childhood caries based on machine learning and compare their effectiveness with the conventional regression model.
Portella 2023 [62], Brazil, *Clinical Oral Investigations*	2481 teeth	VGG19	Retrospective study	There were 8749 photos in the training dataset and 140 images in the test dataset. With an F1 score of 0.887, accuracy of 0.879, precision of 0.949, positive agreement of 0.827, and negative agreement of 0.800, VGG19 performed well. Examiners US, ND, and SP had accuracy rates in Phase I of 0.543, 0.771, and 0.807, respectively. For the same examiners, the accuracy rates increased to 0.679, 0.886, and 0.857 in Phase II. For every examiner, the proportion of right responses was considerably greater in Phase II compared to Phase I (McNemar test; *p* < 0.05).	The aim of this paper was to evaluate the suitability of this deep learning method as a supplemental tool and look into how well a convolutional neural network (CNN) can identify healthy teeth and early carious lesions on occlusal surfaces.
Ramos-Gomez 2021 [63], USA, *Dentistry Journal*	182 patients	Random forest	Retrospective study	The age of the parents (MDG = 0.84; MDA = 1.97), unmet needs (MDG = 0.71; MDA = 2.06), and the child’s race (MDG = 0.38; MDA = 1.92) were among the survey factors that were highly predictive of active caries. Strong predictors of caries experience were the age of the parent (MDG = 2.97; MDA = 4.74), whether the child had experienced dental pain in the previous year (MDG = 2.20; MDA = 4.04), and whether the child had experienced caries in the preceding year (MDG = 1.65; MDA = 3.84).	Using a machine learning algorithm applied to parent survey assessments of their child’s oral health, this study explored the potential of screening for dental caries in youngsters.
Rauf 2023 [64], Iraq, *Medicina*	450 images	k-NN	Prospective study	Following the dataset’s application to both ML algorithms—linear regression and k-nearest neighbors—the evaluation measure reveals that k-NN outperforms LR in terms of prediction accuracy. The accuracy result was close to 99%.	In order to prevent future crowding in patients who are growing or even in adults seeking orthodontic treatment, the study attempted to use artificial intelligence to forecast the arch width as a diagnostic tool for orthodontics.
Seo 2021 [65], Korea, *Journal of Clinical Medicine*	600 radiographs	ResNet-18, MobileNet-v2, ResNet-50, ResNet-101, Inceptionv3, and Inception-ResNet-v2	Retrospective study	More than 90% accuracy was shown by all deep learning models, with Inception-ResNet-v2 demonstrating the best performance.	This work aimed to assess and compare the effectiveness of six cutting-edge deep learning models for cervical vertebral maturation (CVM) based on convolutional neural networks (CNNs) on lateral cephalometric radiographs. Additionally, it visualized the CVM classification for each model through the use of gradient-weighted class activation map (Grad-CAM) technology.
Spampinato 2017 [66], Italy, *Medical Image Analysis*	1391 radiographs	CNN	Retrospective study	The findings demonstrated state-of-the-art performance, with an average difference between manual and automatic evaluation of almost 0.8 years.	In this work, the objective was to present and evaluate multiple deep learning methods for automatically determining the age of skeleton bones.
Tajmir 2019 [67], USA, *Skeletal Radiology*	280 radiographs	CNN	Comparative study	The mean six-reader cohort accuracy was 63.6 and 97.4% within a year, whereas the total AI BAA accuracy was 68.2% and 98.6% within the same time frame. The mean single-reader RMSE was 0.661 years, whereas the AI RMSE was 0.601 years. The combined RMSE dropped from 0.661 to 0.508 years, whereas each RMSE dropped independently with AI support. The ICC was 0.9951 with AI and 0.9914 without.	The aim of this paper was to examine a cohort of pediatric radiologists’ BAA performance both with and without AI support.
Tanikawa 2009 [68], Japan, *Angle Orthodontist*	65 radiographs	CNN	Retrospective study	The algorithm had a mean success rate of 88% (range, 77–100%) in correctly identifying the specified anatomic entities in all the images and determining the landmark placements.	This work set out to assess the reliability of a system that uses landmark-dependent criteria specific to each landmark to automatically recognize anatomic landmarks and surrounding structures on lateral cephalograms.
Toledo-Reyes 2023 [69], Brazil, *Journal of Dental Research*	639 patients	ML algorithms: decision tree, random forest, and extreme gradient boosting (XGBoost)	Prospective study	When it came to predicting caries in primary teeth following a 2-year follow-up, all models’ areas under the receiver operating characteristic curve (AUCs) during training and testing were greater than 0.70, with baseline caries severity being the best predictor. After ten years, the XGBoost-based SHAP algorithm, in reference to the testing set, achieved an AUC greater than 0.70, indicating that the top predictors of caries in permanent teeth were caries experience, nonuse of fluoridated toothpaste, parent education, higher frequency of sugar consumption, low frequency of visits to relatives, and poor parents’ perception of their children’s oral health.	The aim of this research was to use early childhood factors to create and evaluate caries prognosis models using machine learning (ML) in primary and permanent teeth after two and ten years of follow-up.
Vila-Blanco 2020 [70], Spain, *IEEE Transactions on Medical Imaging*	2289 radiographs	DAnet and DASnet	Comparative study	The median absolute error (AE) and median error (E) for the entire database were reduced by around 4 months, demonstrating that DASNet beats DANet in every way. As the real ages of the individuals lowered while analyzing DASNet in the reduced datasets, the AE values declined as well, reaching a median of roughly 8 months in the patients under the age of 15. In comparison to the most advanced manual age estimation techniques, the DASNet method shown notably fewer over- or underestimation issues.	This paper proposed two totally automatic approaches to determine a subject’s chronological age from an OPG image.
Wang 2016 [71], USA, *Medical Physics*	30 patients	Random forest	Retrospective study	Based on manually labelled ground truth, segmentation results on CBCT images of thirty participants were validated, both qualitatively and numerically. The authors’ method yielded average dice ratios of 0.94 and 0.91 for the mandible and maxilla, respectively. These values are significantly higher than those of the state-of-the-art method that relies on sparse representation (*p*-value < 0.001).	The aim of this paper was to incorporate prior spatial information into classification-based segmentation, as this approach has outperformed the reliance on only picture appearances. Additionally, this approach has sought to overcome the difficulties in CBCT maxilla and mandible segmentation, in contrast to the majority of other methods that only concentrate on brain pictures.
Wang 2016 [72], China, *American Journal of Orthodontics and Dentofacial Orthopedics*	88 patients	Eye-tracking device	Comparative study	There were significant differences observed in the scanpaths of laypersons viewing pretreatment smiling faces compared to those of laypersons viewing normal smiling subjects. Specifically, there was less fixation time (*p* < 0.05) and later attention capture (*p* < 0.05) on the eyes, and more fixation time (*p* < 0.05) and earlier attention capture (*p* < 0.05) on the mouth. When comparing post-treatment smiling individuals, similar findings were observed: there was a decrease in fixation duration (*p* < 0.05) and an earlier attention capture on the lips (*p* < 0.05), as well as a decrease in fixation time (*p* < 0.05) and a later attention capture on the eyes (*p* < 0.05). Compared to the normal individuals and post-treatment patients, the pretreatment repose faces showed an earlier attention catch on the mouth (*p* < 0.05). The categorization of pretreatment patients from normal subjects (treatment need) and pretreatment patients from post-treatment patients (treatment result) using a linear support vector machine demonstrated accuracies of 97.2% and 93.4%, respectively.	The aim of this research was to investigate the potential of an eye-tracking technique as a new, impartial means of assessing orthodontic treatment outcomes and need from the viewpoint of the general public, in contrast to more conventional evaluation methods.
Wang 2020 [73], USA, *JDR Clinical & Translational Research*	545 patients	Extreme gradient boosting and naive Bayesian algorithms	Prospective study	The toolkits were created using data from 545 households with children ranging in age from 2 to 17 years old. Using the external data, the prediction accuracies for RFTN were 49% and 93%, respectively. Between the clinically determined COHSI and the anticipated COHSI, there was a correlation of 0.88 (and 0.91 for its percentile). The COHSI toolkit’s RMSEs were 1.3 for its percentile and 4.2 for COHSI.	In order to forecast children’s oral health status index (COHSI) scores and referral for treatment needs (RFTN) assessments for oral health, the aim of this paper was to develop oral health assessment toolkits.
You 2020 [74], China, *BMC Oral Health*	98 images	CNN	Retrospective study	The mean intensity of ulceration (MIoU) on the examined dental images was 0.726 ± 0.165. After a week, the dentist’s MIoU was 0.689 ± 0.253, compared to 0.695 ± 0.269 when diagnosing 98 digital camera photos. The AI model showed a higher MIoU (0.736 ± 0.174) than the dentist, and the results remained the same after a week. The dentist’s and the AI model’s MIoUs after evaluating 102 intraoral pictures were 0.652 ± 0.195 and 0.724 ± 0.159, respectively. A paired *t*-test revealed no statistically significant difference (*p* > 0.05) in the diagnosis of dental plaque on primary teeth between the AI model and the human professional.	The purpose of this work was to develop an artificial intelligence (AI) model based on deep learning to identify plaque on primary teeth and to assess the model’s diagnostic performance.
You 2021 [75], China, *Zhonghua Kou Qiang Yi Xue Za Zhi*	109 images	DeepLab	Prospective study	When training with 440 photographs and testing with 109 photos, the permanent tooth model’s MIoU was 0.700 ± 0.191. The proportion of plaque and the quantity of pixels surrounding the plaque had a significant impact on the accuracy of dental plaque identification in the regression model of the significance test (*p* < 0.05). The standardized coefficients for the percentage of plaque were −0.551 and the number of pixels on the plaque edge line were −0.289.	The aim of this study was to create an AI system that can identify dental plaque on permanent teeth and identify the contributing variables.
Zaborowicz 2021 [76], Poland, *Sensors*	619 images	PNN, GRNN, RBF, and MLP	Prospective study	The study yielded three non-linear models of radial basis function networks (RBFs), with an accuracy range of 96 to 99%, and a collection of 21 unique indicators required for the assessment of chronological age using computer image analysis and neural modeling.	The research presented in this paper aimed to create a new, efficient methodology for the use of contemporary IT techniques in the assessment of chronological age.
Zaborowicz 2022 [77], Poland, *Sensors*	619 images	PNN, GRNN, RBF, and MLP	Prospective study	Depending on the learning set utilized, the generated models’ MAE and RMSE errors ranged from 2.34 to 4.61 months and 5.58 to 7.49 months, respectively. The range of the correlation coefficient (R^2^) was 0.92 to 0.96.	The purpose of this effort was to confirm that a deeper neural network model could be created that was more accurate than models created in the past.
Zaorska 2021 [78], Poland, *Genes*	262 patients	CNN	Prospective study	The logistic regression (LogReg) model had an area under the curve (AUC) of 0.970 (95% CI: 0.912–0.994; *p* < 0.0001), 90% sensitivity, and 96% specificity, with an overall accuracy of 93% (*p* < 0.0001). For the test and validation predictions, the authors discovered 90.9–98.4% and 73.6–87.2% prediction accuracies, respectively. The most powerful predictors were ENAM_rs12640848 (in LogReg), MMP16_rs1042937 (in NN), and AMELX_rs17878486 and TUFT1_rs2337360 (in both LogReg and NN).	The purpose of this work was to develop an artificial neural network-based caries prediction model that used selected SNPs from each of the three studies as predictors.
Zhou 2021 [79], China, *Diagnostics*	1080 radiographs	CNN	Retrospective study	Between human examiners, the mean labelling error was 0.48 ± 0.12 mm. Between AI and human examiners, the mean labelling error was 0.36 ± 0.09 mm. AI results and the gold standard agreed well overall, with an intraclass correlation coefficient (ICC) value of up to 98%. Furthermore, 71% of CVM staging was accurate. The CS 6 stage (85%) had the highest accuracy in terms of F1 score.	The goal of this research was to create an artificial intelligence (AI) system that can assess AI performance and automatically ascertain the CVM state.

**Table 3:** Summary of field of application of AI in pedodontics.

Field of Application	Summary
Orthodontic diagnosis	Patient data is crucial for accurate orthodontic diagnosis in children. Automation, including AI and ML technologies, streamlines evaluations and reduces diagnostic variation. Research suggests AI’s potential to improve orthodontic practices by enhancing accuracy and efficiency, particularly in treatment planning and diagnosis [57,64,72].
Automated cephalometric tracing	In pediatric orthodontics, cephalometry is crucial for diagnosis and treatment planning. Computer-assisted tracing improves accuracy and efficiency compared to manual methods. AI-driven automated tracing achieves high success rates, reducing errors and enhancing time efficiency in orthodontic procedures [35,45,48,49,53,56,58,60,68].
Segmentation and landmark identification	In medical imaging, segmentation identifies organs or lesions crucial for diagnosis and treatment planning. Automated landmark identification in X-rays and orthopantomographs, using CNNs like YOLOv5 and YOLOv4, improves efficiency and accuracy. Deep learning methods also facilitate maxilla and mandibular segmentation in CBCT scans, aiding treatment planning. The ALICBCT algorithm automates landmark identification in CBCT images, enhancing speed and accuracy in orthodontic assessments [22,23,27,39,52,71,80].
AI-driven remote monitoring	Artificial intelligence-driven remote monitoring (AIRM) utilizes smartphones for independent dental scans, allowing clinicians to remotely monitor oral health. While AIRM reduces in-office visits and enhances patient–doctor communication, its cost-effectiveness may vary. Remote monitoring in orthodontic therapy improves patient care and treatment outcomes by enabling frequent assessments and precise diagnosis [33,34].
Estimation of growth and development	In orthodontic treatment, precise timing is crucial, and anthropometric indices like chronological age and skeletal maturation are vital for assessing growth. Skeletal age assessment, often determined through radiographs, is more reliable than relying solely on chronological age. The cervical vertebral maturation (CVM) approach correlates strongly with skeletal maturity assessment. Recent AI advancements automate skeletal age determination, promising improved accuracy and efficiency in orthodontics [21,36,41,43,47,48,55,65,66,67,79].
Dental plaque and cavities	Identifying dental plaque presents challenges, often addressed with time-consuming methods. Recent research utilizes deep learning techniques for plaque detection on primary and permanent teeth, showing promising results but facing challenges in image quality and AI rationale. Another study achieves high accuracy in detecting caries and MIH using digital imaging, suggesting the potential for improved disease detection and clinical judgments [30,62,74,75].
Evaluating pediatric oral health through toolkits developed by machine learning	The WHO created a dental health questionnaire to address disparities, with machine learning tools predicting COHSI and RFTN. The PROMIS framework expands oral health assessment, and a toolkit aids parents in evaluating children’s oral health. Statistical techniques and AI explore the link between teenage quality of life and dental health. The ORIENTATE platform offers machine learning tools for dental health assessments without specialized expertise, revolutionizing outcomes [8,31,73].
Supernumerary tooth identification	Deep learning models assist in diagnosing dental issues like mesiodens and supernumerary teeth, especially when screening is challenging. Models such as ResNet-18 and Inception-ResNet-v2 show promise in detecting mesiodens in primary or mixed dentition, while CNN models like AlexNet and VGG16-TL perform well in identifying supernumerary teeth during transitional dentition. Challenges include limited datasets and difficulties in categorizing panoramic radiography images. AI should complement human expertise, not replace it. Models like DetectNet and AlexNet are promising for recognizing supernumerary teeth, but fully automating diagnosis and determining mesiodens’ quantity and position remain challenges. AI also aids in diagnosing taurodontism with expert-level accuracy. Further advancements are needed for comprehensive diagnostic systems to benefit both dentists and patients [2,11,20,29,32,42,46,54].
Early childhood caries	ML is used for predicting and diagnosing early childhood caries (ECC) and assessing caries risk factors. It identifies genetic markers, compares models like XGBoost and random forest for ECC detection, and analyzes predictors of caries in primary and permanent teeth. ML also examines salivary cystatin S levels and parental surveys for ECC risk assessment. These studies demonstrate ML’s potential in ECC diagnosis and preventive strategies [37,44,59,61,63,69,78].
Chronological age assessment	Various methods, including clinical and pantomographic approaches, assess dental age. Digital orthopantomography accurately estimates age in children (4–15 years). Artificial neural networks and neural modeling techniques predict dental maturity and chronological age in children and teenagers (ages 4–15). Machine learning techniques using radiomorphometric characteristics effectively estimate age. Evaluation of 97 OPT images forecasts age based on the phases of seven permanent teeth using SOS-ResNext50, outperforming existing methods [25,28,51,70,76,77].
Detecting deciduous and young permanent teeth	Various object detection models, including YOLOv3, YOLOv4, Faster R-CNN, and R-CNN, are used in dental disorder identification. CNN-based mapping enhances tooth segmentation accuracy. YOLOv4 excels in tooth detection and counting due to its speed and accuracy. CNN algorithms effectively identify unerupted molars and aid in personalized treatment planning. R-CNN methods swiftly identify and number deciduous and primary teeth in pediatric radiographs, aiding forensic identification. While two-stage detectors offer high accuracy, YOLO stands out for its real-time object recognition and consistent performance [24,26,28,38,40,50].

## Data Availability

Data are available upon request to the corresponding author.

## Supplementary Materials

The following supporting information can be downloaded at https://www.mdpi.com/article/10.3390/healthcare12131311/s1, Table S1: Terms used on database search; Table S2: Articles excluded after full-text evaluation, with reasons (n = 128); Supplementary material S1 contains the protocol registered in OSF.
