# Supplementary material for: The Implications of Artificial Intelligence in Pedodontics: A Scoping Review of Evidence-Based Literature
Source: Healthcare (Basel). 2024 Jun 30;12(13):1311. doi: 10.3390/healthcare12131311 (PMC11240988; doi:10.3390/healthcare12131311)
Supplement: Supplementary file 1 [file healthcare-12-01311-s001.zip › Supplementary Material S1.pdf]

The Implications of Artificial Intelligence in Pedodontics: A Scoping Review of evidence-based literature.

Authors

Salvatore La Rosa, Vincenzo Quinzi, Giuseppe Palazzo, Vincenzo Ronsivalle, Antonino Lo Giudice

Structured summary

Background: Artificial intelligence (AI) has emerged as a revolutionary technology with several applications across different dental fields, including pedodontics. This systematic review has the objective to catalog and explore the various uses of artificial intelligence in pediatric dentistry.

Methods: A thorough exploration of scientific databases was carried out to identify studies ad-dressing the usage of AI in pediatric dentistry until December 2023 in Embase, Scopus, PubMed, and Web of Science databases by two researchers, S.L.R. and A.L.G.

Results: From a pool of 1301 articles, only 64 met the predefined criteria and were considered for inclusion in this review. From the data retrieved, it was possible to provide a narrative discussion of the potential implications of AI in the specialized area of pediatric dentistry. The use of AI algorithms and machine learning techniques has shown promising results in several applications of daily dental pediatric practice, some of them are: 1) assisting the diagnostic and recognizing processes of early signs of dental pathologies, 2) enhancing orthodontic diagnosis by automating cephalometric tracing and estimating growth and development, 3) assisting and educating children to develop appropriate behavior of dental hy-giene.

Conclusion: AI holds significant potential in transforming clinical practice, improving pa-tient outcomes, and elevating the standards of care in pediatric patients. Future directions may involve developing cloud-based platforms for data integration and sharing, leveraging large da-tasets for improved predictive results, and expanding AI applications for the pediatric population.

Introduction: Rationale

Although John McCarthy introduced the term "Artificial Intelligence" (AI) in a 1956 conference, the concept itself goes back to 1943, with a work by McCulloch and Pitts. The goal was to build automated devices, also called machines, capable of carry-ing out human-level tasks in the field of informatics and mathematics (1). While it might be challenging to define, AI is generally understood to refer to a machine pro-gram that is able to think and perform cognitive activities (2, 3). AI is applied in many different fields these days, such as economics, video games, cellphones, healthcare, and the auto industry (4). It's critical to get a solid grasp of fundamental AI terminology in order

to completely appreciate the implications of AI on the subject of pedodontics, reported in Table 1.

Table 1. Explanation of AI terminology.

AI term	Explanation
Artificial Intelligence (AI)	The primary objective of artificial intelligence (AI) is to build intelligent computers that can learn from data and find solutions on their own. When machines are given new information, they may utilize statistical and probabilistic approaches to learn from past examples and make better decisions. Learning, the process by which behavior or performance is enhanced via practice and experience, is a necessary component of intelligent systems (5).<br>AI is being used in medicine to replace the manual standards that were previously used in machine learning (ML) and deep learning (DL) (6).
Machine learning (ML)	Machine learning (ML) use computers to build statistical models and algorithms that improve understanding and reasoning (7). It entails training algorithms on massive datasets to identify patterns, which are subsequently applied to forecast or choose fresh data (8).
Deep learning (DL)	Deep learning (DL) is a subfield of machine learning that uses artificial neural networks to mimic how the human brain learns (9). Because they were trained using a large amount of data and algorithms, they are more accurate (6). Layers of tiny communication units called neurons make up the Artificial Neural Network (ANN), a kind of deep learning. An ANN with several hidden layers is all that deep learning is. A subset of ANN called convolutional neural networks (CNN) is mostly used in general medicine and dentistry (7, 10). CNNs, a subclass of DL, use fully connected layers plus a subsampling layer that resembles a multilayer perceptron to fill an image with visual cortex cells (11).
Big Data	The term "big data" refers to large datasets and/or the compilation of all accessible data from many sources. It may be utilized to identify patterns that result in unique experiences for different individuals (12).

Artificial intelligence has fast gained traction in science and technology. It mostly depends on imaging, which forms the foundation of dentistry. Furthermore, AI is very helpful in evaluating and tracking a patient's health over time, comprehending the long-term effects of medication, and foreseeing potential health risks (10, 13). Artificial Intelligence has the potential to replace the long hours worked by dental practitioners. Moreover, in pedodontics its first usage was to identify cephalometric landmarks in an automatized way, without the human help.

It is also conceivable to integrate healthcare for all, improve people's health at a reduced cost, and deliver individualized, preventative, and predictive dentistry. AI may, above all, raise the bar for dental care by optimizing diagnosis efficacy and ac-curacy, enhancing treatment visualization, simulating results, and forecasting oral health and disorders (7, 10, 13).

Consideration has also been given to utilizing AI models as additional tools to improve the precision and accuracy of diagnostics. Artificial intelligence (AI) has found widespread application in the medical sciences and has demonstrated consid-erable success in various aspects of patient care. This encompasses evaluating a pa-tient's likelihood of falling ill and detecting various diseases (14-16). However, there is a lack of studies in the existing literature that address a comprehensive approach aimed at clinicians, particularly those with a moderate to low level of expertise in this area, in understanding the indications for AI usage in pedodontics and which factors should be considered from diagnosis to the clinical applications. In this regard, scoping reviews are indicated for broader topics when an overall understanding of the size and the scope of previous related research is a priority.

Introduction: Objectives

This scoping review aims to comprehensively investigate the existing body of lit-erature concerning the integration of AI in pediatric dentistry. The focus is on delving into re-cent advancements and the transformative possibilities that this technology holds, with a specific emphasis on its potential to enhance dental health outcomes for children.

Review question

We developed a key research question to guide our search for strategies. The answer to this question could be used by pediatric dentists as a reference: “What are the current developments and challenges in the application of artificial intelligence in pediatric dentistry?”.

Keywords

Artificial Intelligence, AI, pediatric dentistry, pedodontics

PROTOCOL AND REGISTRATION

The present scoping review will follow the guidelines of the Joanna Briggs Institute (JBI)Br for scoping reviews and the Preferred Reporting Items for Systematic Reviews and Meta-Analyses (PRISMA) extensions for scoping reviews (PRISMA-ScR) (17, 18). The protocol of this scoping review is available as Supplementary Material 1 and it is registered on Open Science Framework database (https://osf.io/z6prb).

Eligibility criteria

Participants

Inclusion criteria: original research articles that delve into the application of AI models in the realms of pediatric dentistry and pedodontics

Exclusion criteria: incomplete texts, scoping reviews, narrative reviews, case series, consensus conferences and articles written in languages other than English

Concept

In the present ScR we mapped the scientific literature with the aim to enhance relevance to clinical indications, and the observations were systematically categorized and discussed within distinct domains. These domains were meticulously structured to encompass all the pertinent data extracted from the studies incorporated in the analysis.

Context

This review selectively incorporates original research articles that delve into the application of AI models in the realms of pediatric dentistry and pedodontics.

Information sources

To assess the corpus of current literature on the subject, a few database searches were carried out through December 2023. The development of a search strategy that incor-porated all discovered keywords and free-standing terms was aided by a health scienc-es librarian. The Web of Science, Embase, Scopus, and PubMed databases were utilized. To confirm the validity of every source of evidence listed in the reference list, addition-al research was looked into.

Search

To assess the corpus of current literature on the subject, a few database searches were carried out through December 2023.The outcomes of modifying the approach search for every database are shown in Supplementary Table 1.

Study/Source of Evidence selection

Following the acquisition of search results from every electronic database, the citations were imported into EndNote X9, a reference manager program developed by Clari-vateTM, London, UK. Reports that were duplicates were eliminated, and articles that provided updates or preliminary findings were only assessed once. Two writers, A.L.G. and S.L.R., checked all of the titles and abstracts they had gathered from the databases before reading the full texts of any relevant studies. . The eligibility of the studies was evaluated objectively, and any disagreements were settled after discussion with another author, G.P. The agreement between the reviewers was highly reliable, with a kappa value of 0.963.

Types of Sources

This review selectively incorporates original research articles that delve into the application of AI models in the realms of pediatric dentistry and pedodontics. To maintain focus and rigor, incomplete texts, scoping reviews, narrative reviews, case series, consensus conferences and articles written in languages other than English have all been excluded from consideration, such as studies including adults.

Moreover, there was no restriction in the year of publication of the studies. This meticulous approach ensures a thorough examination of substantive and pertinent research contributions in the specified field.

Data Charting process

In order to gather the characteristics and outcomes (study design, sample size, and objectives) needed for the ensuing literature analysis, two authors (S.L.R. and A.L.G.) created a data extraction form. We had discussed any discrepancies with G.P., another author reviewer. Cohen kappa statistics was employed to evaluate the degree of concurrence between the two authors, with a highly reliable value, 0.963.

Data Analysis and Presentation

The findings derived from the selected papers were presented in a narrative format and through tables, guided by the research question of the Scoping Review (ScR). The reporting approach employed for the results was based on previous works published by other researchers. To better address clinical indications, results were organized and discussed generating specific domains that covered all the information retrieved in the included studies.

Synthesis of results

The methodology employed in reporting the findings of this study is derived from es-tablished frameworks delineated in prior research conducted by other scholars (14, 19). In order to enhance relevance to clinical indications, the observations were systemati-cally categorized and discussed within distinct domains. These domains were meticu-lously structured to encompass all the pertinent data extracted from the studies incor-porated in the analysis. This approach ensures a systematic and comprehensive presentation of the research outcomes, building upon the foundations laid by previous scholarly investigations.

Results: Selection of source of evidence

The reviewers examined 1154 records out of the 1301 citations that the strategy searches had turned up after removing duplicate files. Following the first screening of abstracts and titles, 959 articles were deemed unacceptable. The full texts of 195 of these articles were then obtained for additional review. After a comprehensive exami-nation of the entire texts of those papers, 64 studies were judged appropriate for the review. The publications that were eliminated at this time are included in Supplemen-tary Table 2 along with the reasons given. Figure 1 provides a summary of all study se-lection information. Table 3 contains details of all the included articles.

Characteristics of sources evidence and results of individual sources of evidence

Table 3 contains details of all the included articles.

Synthesis of results

The primary areas of focus for AI models created for use in pediatric dentistry have been: orthodontic diagnosis (n= 3) (20-22), automated cephalometric tracing (n= 9) (23-31), segmentation and and landmark identification (n= 7) (32-38), AI-Driven Remote Monitoring (n= 2) (39, 40), estimation of growth and development (n= 11) (26, 41-50), dental plaque and cavities (n = 4) (51-54), evaluating Pediatric Oral Health through Toolkits Developed by Machine Learning (n= 3) (8, 55, 56), supernumerary tooth identification (n = 8) (2, 11, 57-62), Early childhood Caries (ECC) (n = 7) (63-69), chronological age assessment (n = 6) (70-75), identification of deciduous and young permanent teeth (n = 6) (74, 76-80).

Discussion: Summary of Evidence

The integration of AI technology into dental practices holds the potential to elevate the standard of dental treatment significantly. By providing support to dentists, AI facili-tates optimal dental care, leading to enhanced accuracy in diagnostics, treatment planning, and outcome predictions. Notably, deep learning plays a pivotal role in di-agnosis, promising increased productivity, and improved precision across dental pro-cedures. The advent of readily available data has allowed AI to demonstrate its effica-cy in various pediatric dentistry applications, with Convolutional Neural Network (CNN) models proving particularly effective in expediting and refining patient diag-noses. This collaborative approach encourages active patient engagement, thereby contributing to the overall success rate of dental care (19, 81-83). Figure 2 explores the field of application of AI in pedodontics. Table 4 shows the possible field of application of AI in pedodontics.

Limitations

The lack of global and organized standards for AI development was identified as the main barrier to its efficacy. Furthermore, difficulties with gathering comprehensive data, guaranteeing accessibility, upholding appropriate data structure, and accom-plishing comprehensiveness were noted. (3). However, additional queries are coming up concerning the morality, obligations, value, and application of AI in human life. The main reasons for dissatisfaction among dental practitioners were the challenges of protecting patients' privacy and their unwillingness to adopt AI-based techniques given the need to preserve human interaction in clinical care.

Conclusions

Because it provides trustworthy and efficient solutions across several industries, AI is growing in popularity in the field of pedodontics, as reported in the present review. Subsequent efforts may concentrate on the creation of cloud-based frameworks intended to streamline data integration and encourage cooperative data exchange. Utilizing vast volumes of high-quality data can enhance the accuracy of prediction results and picture interpretation when employing ML techniques, since data is the fundamental building element of robust models. An AI model that has been properly trained may be able to assist in the screening and diagnosis of growing patients in the field of pedodontics research.

Acknowledgements

N/A

Funding

None.

Conflicts of interest

None.

References

1. Tandon D, Rajawat J. Present and future of artificial intelligence in dentistry. Journal of Oral Biology and Craniofacial Research. 2020;10(4):391-6.
2. Mine Y, Iwamoto Y, Okazaki S, Nakamura K, Takeda S, Peng TY, et al. Detecting the presence of supernumerary teeth during the early mixed dentition stage using deep learning algorithms: A pilot study. International Journal of Paediatric Dentistry. 2022;32(5):678-85.
3. Hutson M. AI Glossary: Artificial intelligence, in so many words. Science. 2017;357(6346):19-.
4. Kunz F, Stellzig-Eisenhauer A, Boldt J. Applications of Artificial Intelligence in Orthodontics;An Overview and Perspective Based on the Current State of the Art. Applied Sciences. 2023;13(6):3850.
5. Soegiantho P, Suryawinata PG, Tran W, Kujan O, Koyi B, Khzam N, et al. Survival of Single Immediate Implants and Reasons for Loss: A Systematic Review. Prosthesis. 2023;5(2):378-424.

6. Janiesch C, Zschech P, Heinrich K. Machine learning and deep learning. Electronic Markets. 2021;31(3):685-95.
7. Dave VS, Dutta K. Neural network based models for software effort estimation: a review. Artificial Intelligence Review. 2014;42(2):295-307.
8. Gajic M, Vojinovic J, Kalevski K, Pavlovic M, Kolak V, Vukovic B, et al. Analysis of the Impact of Oral Health on Adolescent Quality of Life Using Standard Statistical Methods and Artificial Intelligence Algorithms. Children (Basel). 2021;8(12).
9. Albayrak B, ÖZdemir G, Us YÖ, YÜZbasioglu E. Artificial intelligence technologies in dentistry. Journal of Experimental and Clinical Medicine (Turkey). 2021;38:188-94.
10. Nguyen TT, Larrivée N, Lee A, Bilaniuk O, Durand R. Use of Artificial Intelligence in Dentistry: Current Clinical Trends and Research Advances. Journal (Canadian Dental Association). 2021;87:l7.
11. Kaya E, Gunec HG, Aydin KC, Urkmez ES, Duranay R, Ates HF. A deep learning approach to permanent tooth germ detection on pediatric panoramic radiographs. Imaging Science in Dentistry. 2022;52(3):275-81.
12. Allareddy V, Rengasamy Venugopalan S, Nalliah RP, Caplin JL, Lee MK, Allareddy V. Orthodontics in the era of big data analytics. Orthodontics and Craniofacial Research. 2019;22 Suppl 1:8-13.
13. Agrawal P, Nikhade P. Artificial Intelligence in Dentistry: Past, Present, and Future. Cureus. 2022;14(7):e27405.
14. Kumar Y, Koul A, Singla R, Ijaz MF. Artificial intelligence in disease diagnosis: a systematic literature review, synthesizing framework and future research agenda. Journal of Ambient Intelligence and Humanized Computing. 2023;14(7):8459-86.
15. Bichu YM, Hansa I, Bichu AY, Premjani P, Flores-Mir C, Vaid NR. Applications of artificial intelligence and machine learning in orthodontics: a scoping review. Progress in Orthodontics. 2021;22(1):18.
16. Bouletreau P, Makaremi M, Ibrahim B, Louvrier A, Sigaux N. Artificial Intelligence: Applications in orthognathic surgery. Journal of stomatology, oral and maxillofacial surgery. 2019;120(4):347-54.
17. Peters MDJ, Marnie C, Tricco AC, Pollock D, Munn Z, Alexander L, et al. Updated methodological guidance for the conduct of scoping reviews. JBI Evidence Synthesis. 2020;18(10):2119-26.
18. Page MJ, McKenzie JE, Bossuyt PM, Boutron I, Hoffmann TC, Mulrow CD, et al. The PRISMA 2020 statement: an updated guideline for reporting systematic reviews. British Medical Journal. 2021;372:n71.
