# Supplementary material for: The Implications of Artificial Intelligence in Pedodontics: A Scoping Review of Evidence-Based Literature
Source: Healthcare (Basel). 2024 Jun 30;12(13):1311. doi: 10.3390/healthcare12131311 (PMC11240988; doi:10.3390/healthcare12131311)
Supplement: Supplementary file 1 [file healthcare-12-01311-s001.zip › Supplementary Table S1.pdf]

Supplementary Table S1. Terms used on database search.

Database	**Search format**
**PUBMED**	("antagonists and inhibitors"[MeSH Subheading] OR ("antagonists"[All Fields] AND "inhibitors"[All Fields]) OR "antagonists and inhibitors"[All Fields] OR "ai"[All Fields] OR ("artificial intelligence"[MeSH Terms] OR ("artificial"[All Fields] AND "intelligence"[All Fields]) OR "artificial intelligence"[All Fields]) OR ("deep learning"[MeSH Terms] OR ("deep"[All Fields] AND "learning"[All Fields]) OR "deep learning"[All Fields]) OR ("machine learning"[MeSH Terms] OR ("machine"[All Fields] AND "learning"[All Fields]) OR "machine learning"[All Fields]) OR ("artif neural netw icann"[Journal] OR ("artificial"[All Fields] AND "neural"[All Fields] AND "networks"[All Fields]) OR "artificial neural networks"[All Fields]) OR (("convolute"[All Fields] OR "convoluted"[All Fields] OR "convolutes"[All Fields] OR "convoluting"[All Fields] OR "convolution"[All Fields] OR "convolutional"[All Fields] OR "convolutions"[All Fields] OR "convolutive"[All Fields]) AND ("neural networks, computer"[MeSH Terms] OR ("neural"[All Fields] AND "networks"[All Fields] AND "computer"[All Fields]) OR "computer neural networks"[All Fields] OR ("neural"[All Fields] AND "networks"[All Fields]) OR "neural networks"[All Fields]))) AND ("paediatric dentistry"[All Fields] OR "pediatric dentistry"[MeSH Terms] OR ("pediatric"[All Fields] AND "dentistry"[All Fields]) OR "pediatric dentistry"[All Fields] OR ("paedodontic"[All Fields] OR "paedodontics"[All Fields] OR "pediatric dentistry"[MeSH Terms] OR ("pediatric"[All Fields] AND "dentistry"[All Fields]) OR "pediatric dentistry"[All Fields] OR "pedodontics"[All Fields] OR "pedodontic"[All Fields]))
**EMBASE via Ovid**	((AI or Artificial intelligence or deep learning or machine learning or artificial neural networks or convolutional neural networks) and (pediatric dentistry or pedodontics)).
**WEB OF SCIENCE**	((AI) OR (Artificial intelligence) OR (deep learning) OR (machine learning) OR (artificial neural networks) OR (convolutional neural networks)) (All Fields) and ((pediatric dentistry) OR (paedodontics)) (All Fields)
**SCOPUS**	(TITLE-ABS-KEY (((ai) OR (artificial AND intelligence) OR (deep AND learning) OR (machine AND learning) OR (artificial AND neural AND networks) OR (convolutional AND neural AND networks))) AND TITLE-ABS-KEY (((pediatric AND dentistry) OR (pedodontics))))
