# Supplementary material for: The Implications of Artificial Intelligence in Pedodontics: A Scoping Review of Evidence-Based Literature
Source: Healthcare (Basel). 2024 Jun 30;12(13):1311. doi: 10.3390/healthcare12131311 (PMC11240988; doi:10.3390/healthcare12131311)
Supplement: Supplementary file 1 [file healthcare-12-01311-s001.zip › Supplementary Table S2.pdf]

Supplementary Table S2. Articles excluded after full-text evaluation, with reasons (n=128)

Article excluded	Reasons for exclusion
(Abu 2019)	4
(Agrawal 2022)	2
(Ajmera 2023)	4
(Al Turkestani 2021)	1
(Al-Namankany 2023)	1
(Alalharith 2020)	4
(Albayrak 2021)	2
(Alevizakos 2022)	4
(Alkhal 2008)	4
(Allareddy 2019)	4
(Almalki 2022)	4
(Anil 2017)	2
(Anthonappa 2012)	4
(Appelstrand 2022)	1
(Aren 2003)	4
(Askar 2021)	4
(Auconi 2011)	4
(Azhari 2023)	4
(Baccetti 2002)	4
(Baccetti 2005)	4
(Baliga 2019)	2
(Bianchi 2020)	4
(Bichu 2021)	2
(Bonny 2023)	2
(Bouletreau 2019)	4
(Broadbent 2009)	4
(Buhmann 2010)	4
(Bulatova 2021)	4
(Cantu 2020)	4
(Carter 2004)	4
(Ceraulo 2020)	4
(Chen 2010)	4
(Chifor 2022)	1
(Cho 2023)	4
(Chu 2023)	4
(Chung 2022)	4
(Dave 2014)	2
(Demirjian 1985)	4
(Evangelista 2022)	1
(Ezhov 2021)	4
(Fan 2022)	4
(Fishman 1979)	4
(Fishman 1982)	4
(Gabriel 2009)	4
(Gandini 2006)	4
(Girshick 2014)	4
(Gunec 2023)	4
(Gurgel 2023)	4
(Hagg 1980)	4
(Hagg 1982)	4
(Han 2022)	4
(He 2015)	4
(Hesamian 2019)	2
(Holtkamp 2021)	4
(Horita 2018)	4
(Humairo 2021)	3

(Hutson 2017)	4
(Hutton 2000)	4
(Janiesch 2021)	4
(Javed 2020)	4
(Jeon 2022)	4
(Joseph 2015)	4
(Kats 2020)	4
(Khanagar 2021)	1
(Khanagar 2022)	1
(Khosravi-Kamrani 2022)	4
(Kim 2009)	4
(Kim 2021)	4
(Korde 2017)	3
(Kumar 2023)	1
(Kunz 2023)	2
(Lahoud 2021)	4
(Leavitt 2023)	4
(Leonardi 2021)	4
(Leonardi 2021)	4
(Li 2021)	4
(Liu 2017)	4
(Liu 2018)	4
(Liu 2022)	4
(Liu 2023)	2
(Lo Giudice 2022)	4
(Maltarollo 2023)	3
(Mason 2023)	4
(McNamara 2018)	4
(Mito 2002)	4
(Mohamed 2021)	4
(Morris 2012)	4
(Murata 2017)	3
(Navlani 2013)	4
(Nguyen 2021)	4
(Okazaki 2022)	4
(Olszowski 2012)	4
(Page 2021)	4
(Patcas 2019)	4
(Peng 2020)	4
(Prasad 2022)	4
(Proffit 2018)	3
(Qazi 2023)	4
(Qu 2022)	4
(Reddy 2022)	4
(Ren 2015)	4
(Rohrer 2022)	4
(Ruiz 2023)	4
(Sagel 2000)	4
(Schlickenrieder 2021)	4
(Schneider 2022)	4
(Schwendicke 2019)	2
(Schwendicke 2021)	4
(Schwendicke 2021)	4
(Schwendicke 2022)	4
(Schwendicke 2022)	4
(Seo 2023)	4
(Silva 2018)	4
(Soegiantho 2023)	1
(Szemraj 2018)	1

(Tandon 2020)	4
(Tiwari 2023)	1
(Tiwari 2023)	1
(Tuzoff 2019)	4
(Vishwanathaiah 2023)	1
(Volgenant 2016)	4
(Wong 2023)	2
(Wu 2022)	4
(Xiao 2021)	4
(Yagi 2010)	4
(Zhao 2012)	4
(Zheng 2021)	4
(Zhu 2022)	4

Reasons for exclusion: 1-Systematic Review or narrative review article; 2-Review or scoping review; 3- Interview/editorial/book title/ conference; 4- Topic not compatible with the subject of the study;
